# Chromosome Pairing in Polyploid Grasses

**DOI:** 10.3389/fpls.2020.01056

**Published:** 2020-07-09

**Authors:** Radim Svačina, Pierre Sourdille, David Kopecký, Jan Bartoš

**Affiliations:** ^1^ Institute of Experimental Botany of the Czech Academy of Sciences, Centre of the Region Haná for Biotechnological and Agricultural Research, Olomouc, Czechia; ^2^ INRA, Génétique, Diversité, Ecophysiologie des Céréales, Clermont-Ferrand, France

**Keywords:** chromosome pairing, homoeologous pairing, meiosis, Poaceae, polyploidy

## Abstract

Polyploids are species in which three or more sets of chromosomes coexist. Polyploidy frequently occurs in plants and plays a major role in their evolution. Based on their origin, polyploid species can be divided into two groups: autopolyploids and allopolyploids. The autopolyploids arise by multiplication of the chromosome sets from a single species, whereas allopolyploids emerge from the hybridization between distinct species followed or preceded by whole genome duplication, leading to the combination of divergent genomes. Having a polyploid constitution offers some fitness advantages, which could become evolutionarily successful. Nevertheless, polyploid species must develop mechanism(s) that control proper segregation of genetic material during meiosis, and hence, genome stability. Otherwise, the coexistence of more than two copies of the same or similar chromosome sets may lead to multivalent formation during the first meiotic division and subsequent production of aneuploid gametes. In this review, we aim to discuss the pathways leading to the formation of polyploids, the occurrence of polyploidy in the grass family (Poaceae), and mechanisms controlling chromosome associations during meiosis, with special emphasis on wheat.

## Introduction

Poaceae (grasses) is a large family of monocotyledonous flowering plants that includes ~10,000 diverse species divided into 12 subfamilies, 51 tribes, and 80 subtribes (Soreng et al., 2015). This family includes the cereals, bamboos, as well as natural and cultivated grasses, and its members are found worldwide except in ice-covered areas. Their economic importance derives mainly from their utilization for food and feed production, but they also have ecological and aesthetic roles in ecosystems and for humanity. For example, maize (*Zea mays*), rice (*Oryza sativa*), and wheat (*Triticum aestivum*) together provide >50% of the calories consumed by all humans. Sugarcane (*Saccharum officinarum*) remains the major source of human-consumed sugar and is increasingly used for biofuel production. Ryegrasses (*Lolium* spp.), fescues (*Festuca* spp.), and bluegrasses (*Poa* spp.) are cultivated as fodder crops and for amenity purposes (i.e. sports, private and industrial lawns). Bamboos (Bambuseae) are used to construct elaborate scaffolds and the straws of cereals can serve as insulation in buildings or as raw material for paper production. All these uses make the Poaceae species a priority choice for enhancing both their quality (i.e., protein, lipid or sugar contents; cooking-quality, and digestibility, among others) and quantity (yield of grain and straw, biomass production).

Besides their great economic importance, species of the Poaceae family also serve as excellent model organisms for evolutionary studies (Kellogg, 2001). According to the pollen fossil record, grasses arose 55–70 million years ago (MYA; Jacobs et al., 1999). With ever more sequenced genomes (for details see https://bioinformatics.psb.ugent.be/plaza/), a detailed investigation of the evolutionary fate of duplicated chromosomal blocks led to the proposition of an ancestral karyotype for grasses, one structured in seven protochromosomes that contained 16,464 protogenes (Murat et al., 2014). This ancestral genome then further evolved, through the fusion and fission of chromosomes, gene duplication events as well as deletions, and chromosomal inversions and translocations. Moreover, interspecific hybridization and polyploidization (whole genome duplication; WGD) are two other key mechanisms of speciation in the Poaceae. All these phenomena have contributed to the extensive genome diversity extant within the family, including its variability in basic chromosome numbers and a wide range of polyploidy levels (Keeler, 1998). In this review, we highlight the nature of polyploidy in grasses, using wheat as a model, with a special focus on chromosome pairing during meiosis.

## Polyploidy

Polyploidy plays a significant role in the evolution of higher plants, in that all angiosperms apparently underwent at least one round of WGD in their evolutionary history (Jiao et al., 2011). Polyploids can be categorized based on their origin. *Autopolyploids* possess three or more copies of the same chromosome set; by contrast, the multiple chromosome sets in *allopolyploids* are of different origin, due to the involvement of interspecific hybridization. Yet a strict boundary between these two categories is not always evident, such that a third (intermediate) group called segmental allopolyploidy is sometimes recognized in plants (Winterfeld et al., 2012). In general, autopolyploids often exhibit the formation of multivalents during meiosis and polysomic inheritance in their progeny. By contrast, allopolyploids with distant parental genomes usually exhibit formations of bivalents from homologous chromosomes (i.e., diploid-like pairing behavior), leading to disomic inheritance (Ramsey and Schemske, 1998). Nevertheless, allopolyploids sometimes carry chromosome sets that are not identical, but divergence of their sequence is insufficient to avoid the pairing of homoeologs (i.e., chromosomes originating from two related parental genomes with substantial homology); hence, they must employ an additional mechanism to ensure diploid-like behavior. Jauhar (2003) suggested that stable meiotic behavior and genome stability in allopolyploid species is achievable only after establishing a mechanism to ensure homologous chromosome recombination and segregation.

### Autopolyploids

For a long time, autopolyploids were believed to suffer from various evolutionary disadvantages, leading to the conviction that autopolyploidy is rare in nature and often represents an evolutionary dead end (Clausen et al., 1945; Stebbins, 1971). This view, however, contrasts with their widespread utilization in crop production, for which many autopolyploids including potato, banana, watermelon, and sugarcane are of high economic importance. The proportion of autopolyploidy among plant species can only be debated so far, given that many autopolyploids have escaped recognition, being morphologically similar to their progenitors and concealed among common diploid taxa (Soltis et al., 2007). Recently, Barker et al. (2016) inferred that autopolyploids might be as frequent as allopolypoids among vascular plants. The Poaceae family contains many known autopolyploid species, such as *Andropogon gerardii*, a dominant grass of the tallgrass prairie (Keeler and Davis, 1999), several *Brachiaria* species (Gallo et al., 2007), the forage crop *Hordeum bulbosum* (Eilam et al., 2009), the sugarcane plant *S. spontaneum* (Wang et al., 2010), in addition to several *Avena* species (Ladizinsky, 1973).

### Allopolyploids

Allopolyploids result from the hybridization of two more or less related species, such as *Psidium guineense* (Marques et al., 2016), wheat (*T. aestivum*) or the common oat (*Avena sativa*). Genomes inherited by allopolyploids vary in chromosomal homology, based on congeniality of parental species. In the case of hybridization between distantly related species, chromosomal homology can be low enough to not pair up during meiosis, frequently having different basic number of chromosomes. Conversely, allopolyploids that originated from the cross between closely related species carry chromosomes with much higher degree of homology. Accordingly, their homoeologous chromosomes have the potential to pair and recombine during meiosis (Ramsey and Schemske, 1998; Sun et al., 2017). Bread wheat is a typical example of an allopolyploid; it originated from two distinct interspecific hybridizations among three related diploid species that diverged 5–7 MYA (Marcussen et al., 2014). The first hybridization event occurred <0.82 MYA, between *T. urartu* and an as of yet unknown species from the *Sitopsis* section, closely related to *Aegilops speltoides*, which resulted in the development of a tetraploid species that further evolved into cultivated tetraploid wheat (*T. turgidum* ssp. *durum*; BBAA; Marcussen et al., 2014). The second hybridization took place more recently, between this newly developed tetraploid and *Ae. tauschii* (DD), resulting in hexaploid *T. aestivum* (2n = 6*x* = 42; BBAADD; Huang et al., 2002; Petersen et al., 2006; Marcussen et al., 2014). Similarly, oats (*Avena* spp.) also comprise diploid, tetraploid, and hexaploid species, either as auto- or allopolyploids. The allopolyploid oats behave diploid-like during meiosis despite having partial homology between their parental genomes (Thomas, 1992). Besides evolutionarily old allopolyploids, relativey recent allopolyploidazion events are evident in nature. For example, about 150 years ago, the two natural hybrids *Spartina × neyrautii* and *S. × townsendii* emerged through crosses between European *S. maritima* and *S. alternifolia*, the latter introduced from America. While the homoploid hybrid *S. × townsendii* is mostly sterile, chromosome doubling gave rise to the fertile allotetraploid species *S. anglica* (Hubbard, 1968) which spread rapidly throughout salt marshes in Western Europe (Gray et al., 1990; Thompson et al., 1991; Baumel et al., 2001; Salmon et al., 2005). As such, the polyploidization found in *S. anglica* may represent a way by which interspecific hybridization can foster evolutionary success.

### Pathways Leading to Polyploidy

**Figure 1:**
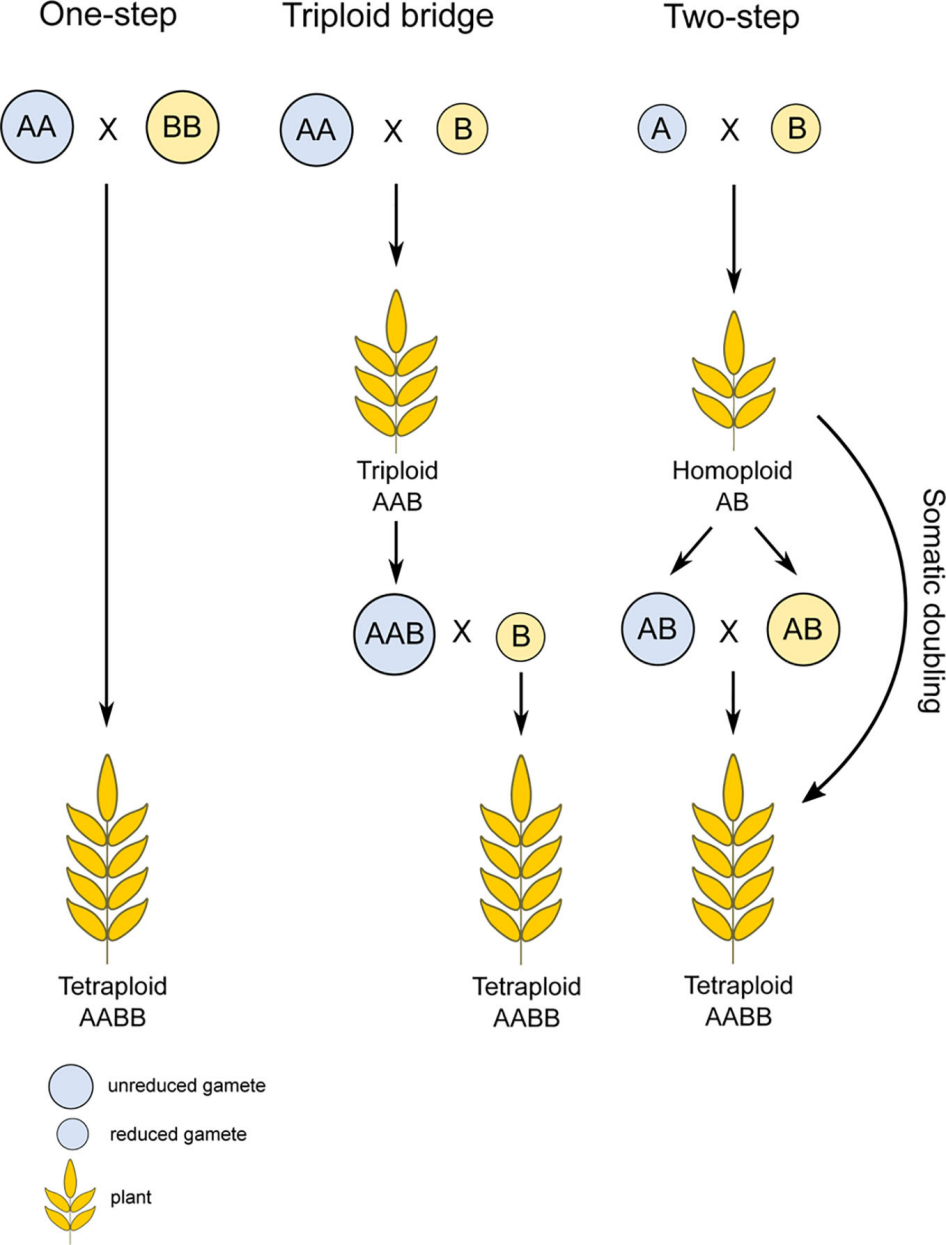
Possible pathways of allopolyploid formation. Polyploidy can be achieved *via* multiple ways, most often through unreduced gamete formation and subsequent fertilization. In the case of the one-step pathway, two unreduced gametes merge, resulting directly in a polyploid species. Arguably, however, more steps are usually needed, where the reduced gamete merges with an unreduced gamete, forming a triploid bridge that requires an additional reduced gamete in subsequent generations. The final depicted option is the two-step pathway, through a homoploid hybrid, which needs a somatic doubling event or unreduced gamete formation to attain a polyploid state.

There are several routes leading to the formation of a polyploid individual. The first way is *via* chromosome doubling because of non-disjunction during mitosis. However, this way is rarely observed under natural conditions and is usually achieved only by exposure to chemical agents (Ramsey and Schemske, 1998; Tamayo-Ordóñez et al., 2016; Pelé et al., 2018). The more likely mechanism operating is that through the generation of unreduced gametes. The frequency of their production usually varies from 0.1% to 2% (Kreiner et al., 2017; Pelé et al., 2018) but this increases in response to stress, such as drought, low or high temperatures, and physical damage (Mason et al., 2011; Pécrix et al., 2011; De Storme et al., 2012; Vanneste et al., 2014; Kreiner et al., 2017; Van de Peer et al., 2017). This fact indicates polyploid formation could accelerate in periods of intensive environmental disturbances and rapid changes (Soltis et al., 2007). Polyploidy can be achieved in a single step process by fusing two unreduced gametes, through a so-called triploid bridge, or *via* a pathway involving two steps (**Figure 1**). The triploid bridge is expected to more commonly occur than the one-step pathway, due to the low probability of fusion of two unreduced gametes in natural populations (Husband, 2004). The two-step pathway of allopolyploid formation first involves generation of a homoploid hybrid. Such an individual would either require a somatic doubling event, fusion of its two unreduced gametes, or involvement of the triploid bridge to restore its fertility (Mason and Pires, 2015). Alternatively, when the progenitors are autopolyploids, an allopolyploid can emerge immediately through the fusion of their standard (i.e., reduced) gametes (Pelé et al., 2018).

Polyploid species usually revert to a diploid state during evolution. The first part of this process, called *cytogenetic* *diploidization*, results in the formation of species, whose polyploid origin might be hidden by disomic inheritance and diploid-like meiosis. This step occurs rather rapidly after polyploid formation either by establishment of genetic control mechanism similar to Ph system in wheat (see below) or extensive chromosomal rearrangements. Over millions of years *genomic diploidization* continues. The content of the genes, which has doubled by polyloidization, is gradually returned towards one copy for each gene. For example, maize underwent an ancient WGD ~10 MYA. Since then, it has not only become cytogenetically diploid but also undergone extensive gene loss causing many genes to revert to a single-copy status in the genome (Renny-Byfield et al., 2017).

### Advantages and Risks of Polyploidization

The question still stands: what is the main evolutionary advantage of polyploid formation in plants? While it may appear to have little impact on particular species (Meyers and Levin, 2006), it can also represent a significant evolutionary tool for improving possibilities of adaptation (Otto and Whitton, 2000). For example, gene redundancy offers an opportunity to better resist deleterious mutations and to diversify the extra copies of genes in subsequent evolution; in this way, new traits may be acquired without the adverse effects of losing the original genes’ function (Ha et al., 2009). From comparative analysis of collinear genes in syntenic regions of wheat and its diploid relatives Akhunov et al. (2013) confirmed the increased gene diversification conferred by polyploidy. Besides gene redundancy, allopolyploids can also benefit from the advantages of heterosis immediately upon their formation (Osborn et al., 2003; Comai, 2005), which can foster a greater biomass and accelerated development. Similarly, autopolyploidy might result in higher biomass of plants (Stebbins, 1971) and seed size, the latter enabling a more rapid rate of early development, such as in *Triticum* and *Aegilops* species (Villar et al., 1998; von Well and Fossey, 1998). All these effects of polyploidization could contribute to faster colonization of new niches, including extreme habitats (Ehrendorfer, 1980). At the chromosomal level, the existence of extra chromosomal set(s) represents a significant fitness advantage for tolerating large rearrangements in the genome that would normally lead to fatal consequences in diploid progenitors.

Clearly then, polyploid species are evolutionarily successful. In many cases (e.g., *T. aestivum*) they can grow in broad geographical areas and occupy a range of habitats (Feldman and Levy, 2005; Dubcovsky and Dvorak, 2007) as well as colonize extreme environments, like *S. anglica* has done (Hubbard, 1968; Gray et al., 1990; Thompson et al., 1991; Baumel et al., 2001; Salmon et al., 2005). Van de Peer et al. (2009) argued the higher competitiveness of polyploids could be explained by an ability to produce more diverse phenotypes than diploid species. Finally, it is worth noting that many staple crops are in fact polyploid species, and humankind has been using artificial polyploidization techniques and wide hybridization as a tool for their breeding and crop improvement. The use of wild relatives to enhance crops dates back to the early 1940s but gained prominence during the 1970s and 1980s (Hajjar and Hodgkin, 2007). Specifically, allopolyploidization is implemented to widen the target species’ genetic diversity or to introgress beneficial alleles from relatives into cultivated crops. For example, while the natural genetic diversity of elite sown material is significantly lower than that observed in its landraces, breeding programs have introduced new sources of diversity into wheat’s cultivars. To date, novel alleles have been introgressed from more than 50 related species representing 13 genera, highlighting the importance of these alien introgressions for improved wheat breeding (Wulff and Moscou, 2014). Perhaps the most well-known case is the rye (*Secale cereale*) 1RS translocation that harbors genes involved in a plant’s resistance to multiple diseases (*Pm8*/*Sr31*/*Lr26*/*Yr9*) and its yield enhancement. Other examples of introgressions include that of *Sr36*/*Pm6* from *T. timopheevii*, *Lr28* from *Ae. speltoides*, and *Pch1* and *Sr38*/*Lr37*/*Yr17* from *Ae. ventricosa*, which provided resistance to severe diseases such as stem and leaf rust and powdery mildew. Some of these introgressions were implemented gobally in commercial lines; for example, the 1RS.1BL translocation now found in 10% of the world’s genetic wheat diversity (Balfourier et al., 2019).

Nontheless, in addition to its positive impacts, polyploidy may have negative aspects. Perhaps the most obvious issue is the presence of more than one pairing partner in meiosis. Unless it is properly processed, it could result in multivalent formation and the production of aneuploid gametes, and thus, lower fertility or complete sterility (Ramsey and Schemske, 2002). Among the adaptive mechanisms described for autopolyploids, there is one based on a reduction in the number of cross-overs to one per chromosome pair, thereby ensuring only bivalents form from any two random homologs (Lloyd and Bomblies, 2016). This mechanism was observed in natural accessions of autotetraploid *Arabidopsis arenosa* (Carvalho et al., 2010; Pecinka et al., 2011; Yant et al., 2013; Pelé et al., 2018). By contrast, recognition of homologous chromosomes is critical for diploid-like pairing in allopolyploids. In allopolyploids containing distinct genomes, it is usually maintained by sequence variation between homoeologous chromosomes. In allopolyploids containing closely-related genomes, homolog recognition seems to be genetically controlled (Jenczewski and Alix, 2004). However, some allopolyploid and homoploid hybrids do not necessarily display significantly reduced fecundity, despite the pairing of homoeologous chromosomes. In such case, aneuploidy, chromosome rearrangements, and the predominance of one of the parental genomes could be observed, as described for ×Festulolium hybrids (Kopecký et al., 2006). Hereon, we focus on mechanisms controlling chromosome pairing in some crops belonging to the grass family (Poaceae).

## Control of Chromosome Pairing in Polyploid Grasses

Meiosis is a crucial process for sexual reproduction and gamete formation. It ensures reduction of genetic material to half resulting in restoration of normal chromosomal constitution in progeny. As noted above, some allopolyploids have evolved molecular mechanisms that govern homologous chromosome pairing. Such regulators were observed and identified in several species, including those of *Triticum*, *Avena*, and *Festuca*. The origin of the genes responsible for regulating chromosome pairing is not known yet, however. Nonetheles, several hypotheses explaining the possible emergence of such mechanisms have been proposed.

The first hypothesis works by presuming the presence of these pairing regulators in diploid progenitors (Waines, 1976; Jenczewski and Alix, 2004). In this model, a stable allopolyploid would emerge after a rare event, in which the appropriate combination of such genes is achieved (Waines, 1976). Indeed, several regulators acting as suppressors of homoeologous chromosome pairing were believed to exist in diploid relatives of allopolyploids, such as *Lolium* spp., *Hordeum* *vulgare* (Gupta and Fedak, 1985), *Hirschfeldia* *incana* (Eber et al., 1994), *Secale* *cereale* (Riley and Law, 1965), *Elytrigia* *elongata* (Dvorak, 1987), *Triticum* *monococcum* (Shang et al., 1989), and *Ae.* *tauschii* (Attia et al., 1979). In *Lolium*, the pairing suppressors were found present in some accessions of *L. multiflorum* and *L. perenne*, where they influenced the number of chiasmata during the first meiotic division of their homoploid hybrid. This chiasma reduction was accounted for exclusively by homoeologous pairing, as revealed by artificially tetraploidized hybrids (Evans and Aung, 1985; Jenczewski and Alix, 2004). Another example of how chromosome-pairing control is induced through a combination of genotypes or genes was found in rice. Generally, rice intersubspecific autotetraploid hybrids display meiotic instability such as chromosome lagging and the formation of univalents and trivalents (Cai et al., 2007). Yet two lines PMeS-1 and PMeS-2 were distinguished as being stable, presumably due to the presence of one or more active meiotic regulator *PMeS* (polyploid meiosis stability) genes (Cai et al., 2007). These two lines display regular meiotic behavior, with bivalents and quadrivalents. The existence of genetic chromosome pairing *PMeS* control was confirmed by the persistent meiotic stability of the two lines even after several generations (Xiong et al., 2019).

The second hypothesis posits that the regulators of chromosome pairing emerge during or immediately after the formation of polyploids, by a mutation or multiple, successive mutations (Riley and Law, 1965; McGuire and Dvořák, 1982). This can happen *via* conversion of a gene that promotes chromosome pairing in the diploid progenitor into a repressor in the polyploidy individual (Riley and Kempanna, 1963; Feldman, 1966b). This phenomenon was described in hexaploid wheat, where a mutation in a pairing promoter gene on the long arm of its chromosome 5D caused a reduction of homoeologous chromosome paring in several interspecific hybrids. Such mutations provide a more pronounced effect than does being 5D nullisomic, which suggests the mutation is antimorphic, changing the gene’s function from pairing-promotion to suppression (Viegas et al., 1980). Those authors argued that this allele more likely arose from spontaneous mutation of a pairing-promoter known to be located on 5DL than from the transfer of *Ph1* from chromosome 5B.

The third hypothesis proposes that such regulators of chromosome pairing could be transferred *via* accessory B chromosomes (Riley et al., 1973; Sears, 1976). Early allopolyploid species would have depended on the presence of a B chromosome(s), until the gene was transferred to an A chromosome by translocation, with the subsequent loss of the B chromosome from the karyotype (Jenczewski and Alix, 2004). Many studies have investigated the role of B chromosomes in the repression of homoeologous pairing (Evans and Macefield, 1972; Evans and Macefield, 1973; Aung and Evans, 1985). It seems that one or more B chromosomes from a specific source could complement one copy of the aforementioned homoeologous-pairing suppressor into a functional complex. Evans and Aung (1986) found homoeologous pairing dramatically reduced in the hybrids of *F. arundinacea* × *L. perenne* carrying B chromosomes. Also, the average number of chromosome arms joined by chiasmata is reduced in the presence of B chromosomes in a diploid meadow fescue when compared to the control plants lacking B chromosomes (Kopecký et al., 2009). In the hybrids of *Ae. mutica* and *Ae. speltoides*, the B chromosomes can also complement a missing *Ph1* locus (Dover and Riley, 1972). Mechanisms controling chromosome pairing in allopolyploids seems to be specific among individual taxa, with very little known of the molecular pathways contributing to this phenomenon. In this respect, the best-elucidated molecular mechanism concerning the *Ph* genes is that of hexaploidy wheat (*T. aestivum*), which we describe in greater detail later on.

Apart from specific genetic systems to ensure proper chromosome pairing in particular species, various other (more general) genes are involved during process of meiosis that could increase the frequency of cross-overs between homologous chromosomes while suppressing them between homoeologs. Recently, Gonzalo et al. (2019) studied the effect of *MSH4* upon homo- and homoeologous cross-overs, by using the EMS (ethylmethanesulphonate) mutant population in *Brassica napus*. They discovered that, when the *MSH4* gene returns to a single copy status, the frequency of homologous cross-overs remained at the same frequency, whereas that of homoeologous cross-overs decreased drastically compared with the presence of two functional copies of the gene. Gonzalo et al. (2019) also studied the copy numbers of other genes of the synapsis-initiation complex (SIC, or alternatively ZMM-pathway) vis-à-vis diploid relatives, deducing that the acquisition of additional copies of such genes through small-scale duplications is a rare event; an example its occurrence is *ZIP4* in wheat (Rey et al., 2017). Furthermore, the rapid reduction in the number of copies for ZMM genes in many species after whole genome duplication—namely for *MSH4*, *MSH5*, *MER3*, and *ZIP4*—supports the hypothesis that ensuring fewer copies of such genes could be a general process of meiotic stabilization (Lloyd et al., 2014; Gonzalo et al., 2019). Another study found no evidence for an increased loss of those genes after polyploidization in hexaploid wheat (including *MSH4*), in that most meiotic genes were retained in three homoeologous variants at similar expression levels (Lloyd et al., 2014). However, because wheat underwent its two hybridization events rather recently (Marcussen et al., 2014), the potential ZMM pathway gene reduction cannot be ruled out. Alternatively, the machinery established *via Ph* genes might have weakened the selective pressure for fewer copies of these genes.

### Chromosome Pairing in Wheat

Allohexaploid bread wheat (*T. aestivum* L.; 2n = 6*x* = 42; BBAADD) can serve as a model plant for meiotic behavior analyses of allopolyploids. Despite the coexistence of three highly similar genomes, its meiotic behavior is strictly diploid-like, with 21 bivalents between homologous chromosomes forming in metaphase I of meiotic division. It has been known for more than 60 years that bread wheat developed genetic control of precise formation of homologous chiasmata, which is enforced by *Ph* (pairing homoeologous) genes (Sears and Okamoto, 1958; Riley and Chapman, 1958). The hexaploid nature of wheat allowed for the development of various aneuploid stocks, permitting the identification of several key genes involved in the regulation of meiosis (Sears and Okamoto, 1958; Sears, 1976; Sears, 1977; Sears, 1982; Sears, 1984).

It was proposed that premeiotic chromosome associations in interphase nucleus also play role in homolog recognition (Brown and Stack, 1968; Comings, 1968; Loidl, 1990; Aragón-Alcaide et al., 1997; Schwarzacher, 1997; Mikhailova et al., 1998; Martínez-Pérez et al., 1999). Nevertheless, different studies disagree in the extent and role of premeiotic chromosome associations, where they start and how long they last (Schwarzacher, 1997; Mikhailova et al., 1998; Martínez-Pérez et al., 1999). However, all these studies partially agree with Feldman (1966a), who suggested that *Ph1* controls spatial organization of chromosomes in premeiotic interphase nuclei. In wheat, the arrangement of chromosomes in interphase nuclei is done through distribution of centromeres and telomeres in opposite sides of nuclei into Rabl configuration (Fussell, 1987), whereas this configuration is being maintained in premeiotic cells (Naranjo, 2015). This organization plays a role in the recognition of homologs, as it reduces the homolog search and simplifies the subsequent alignment (Pernickova et al., 2019). The telomeres are then recruited to the nuclear envelope and form a telomere bouquet (Dawe, 1998; Harper et al., 2004), which is believed to be essential for homolog identification and initiation of synapsis (Bass et al., 2000; Scherthan, 2001; Bass, 2003; Harper et al., 2004; Scherthan, 2007). The molecular mechanisms driving these changes are, however, mostly unknown.

Formation of chiasmata in wheat is driven by both suppressors and promoters, of which several have already been identified. The most important gene regulating homologous chiasmata is *Ph1* (*Pairing homoeologous 1*), located on the long arm of chromosome 5B (Sears and Okamoto, 1958; Riley and Chapman, 1958). Another gene affecting chromosome behavior during meiosis, called *Ph2*, is located on the short arm of chromosome 3D but it exerts a weaker effect than does *Ph1* (Mello-Sampayo, 1971). The least effective regulator, *Ph3*, is located on the short arm of chromosome 3A (Driscoll, 1972; Mello-Sampayo and Canas, 1973). Similar effects of *Ph2* and *Ph3* genes and their location on the same chromosomes of different parental genomes suggest these two genes are probably paralogs. During metaphase I of meiosis, *ph* mutants typically display fewer ring bivalents (with two or more chiasmata) and more univalents, rod bivalents and multivalents when compared to the wild type (**Table 1**).

**Table 1:** Comparison of chromosome associations in hexaploid and tetraploid wheat plants and particular *ph* mutants during metaphase I (Martínez et al., 2001a; Martínez et al., 2001b).

Genotype	Chromosome number	Univalents	Rod bivalents	Ring bivalents	Multivalents	Chiasmata per cell
Hexaploid WT	42	0.02	1.48	19.50	0.00	40.49
*ph1b*	42	2.76	4.76	14.5	0.77	38.57
*ph2b*	42	0.48	2.95	17.78	0.00	34.22
Tetraploid WT	28	0.04	0.34	13.64	0.00	27.62
*ph1c*	28	0.94	3.69	9.46	0.19	23.16

#### *Pairing Homoeologous 1 (Ph1)*

Among those genes controlling chiasmata formation during meiosis in wheat, *Ph1* has the strongest effect on ensuring the correct recognition of homologous chromosomes. Although the presence of this control element was discovered over 60 years ago, its molecular effect was uncovered in part only recently. Its existence was first proposed by Sears and Okamoto (1958) and Riley and Chapman (1958) in haploid lines of wheat lacking chromosome 5B, in which the formation of both bivalents and trivalents had been observed. This contrasted with the meiotic behavior of lines carrying a copy of 5B. Subsequent gene mapping was carried out using the *Ph1* mutant called *ph1b* (Sears, 1977), which helped to delimit the gene’s location. Later mapping, by Gill et al. (1993), used deletion lines to narrow down the genome region harboring the gene, which was cytogenetically estimated to be ~70 Mb. A more recent estimate of this deletion’s length put its at 54.6 Mb (Gyawali et al., 2019). Countless studies have shown that when *Ph1* is missing, the chiasmata formation is no longer strictly diploid-like and chromosomes will form multivalents in more than 50% of pollen mother cells (Riley and Chapman, 1958; Riley, 1960). Work by Sánchez-Morán et al. (2001) confirmed that stark irregularities, such as aneuploidy and genomic rearrangements, are observable in lines lacking *Ph1*.

**Figure 2:**
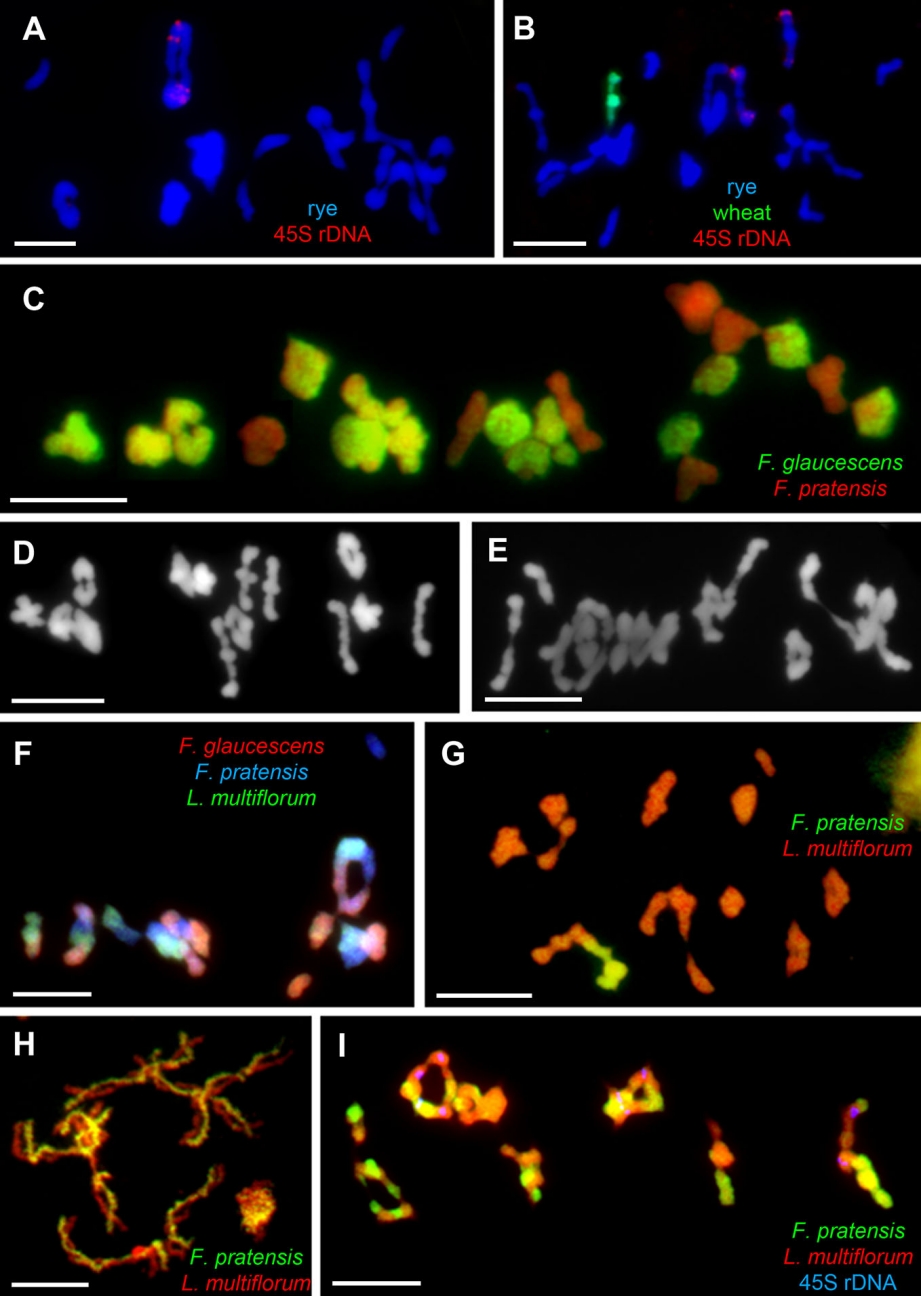
Chromosome assocaitions in allo- and autopolyploids from the Poaceae family. Chromosome pairing in autotetraploid rye (2n = 4*x* = 28, RRRR) differs depending on the presence or absence of *Ph1* located on the introgressed 5BL chromosome arm of wheat. In **(A)**, trivalents and quadrivalents are commonly observed in the control line (2I+4II+2III+3IV), in **(B)**, multivalent chromosome formation is reduced in the line (6I+7II+2IV), where 5B and 5BL are introgressed. In both **(A, B)**, genomic DNA of *Triticum aestivum* was labeled with digoxigenin (green coloring), 45S rDNA was labeled with biotin (red), and genomic DNA of *Secale cereale* served as blocking DNA; all chromosomes counterstained with DAPI (blue). In **(C)**, the chromosome-pairing control system similar to that of *Ph1* found in allohexaploid *Festuca arundinacea* (2n = 6*x* = 42) hampers the associations of homeologous chromosomes and multivalent formation (21II). Genomic DNA of *F. glaucescens* was labeled with digoxigenin (green), while genomic DNA of *F. pratensis* was used as blocking DNA; all chromosomes were counterstained with DAPI (red pseudocolor). In **(D)**, the homoeolog suppressor was probably inherited from one of the progenitors, *F. glaucescens*, as this species also forms only bivalents during meiosis (14II). Conversely, in **(E)**, multivalent formation was detected in the autotetraploid form of the other progenitor, *F. pratensis* (2I+7II+3IV). The system is hemizygous-ineffective, thus allowing for promiscuous homeologous chromosome associations in tetraploid hybrids of *F. arundinacea* × *Lolium multiflorum*, where only one copy of the gene(s) is present **(F)**. Here, genomic DNA of *F. glaucescens* was labeled with biotin (red coloring) and that of *L. multiflorum* labeled with digoxigenin (green), while that of *F. pratensis* was used as blocking DNA; all chromosomes were counterstained with DAPI (blue). In **(G)**, homeologous chromosomes of *F. pratensis* and *L. multiflorum* pair freely in the substitution lines (1I+8II+1III+2IV) as well as in diploid *Festuca* × *Lolium* hybrids (7II), as seen in diplotene shown in **(H)**, due to the absence of any chromosome pairing system and the phylogenetic relationship of both genomes. Note many chiasmata between homeologous chromosomes. This results in frequent homeologous recombinations and massive chromosome rearrangements in successive generations **(I)**, as can be seen in the tetraploid *L. multiflorum* × *F. pratensis* cv. ‘Sulino’ (7IV). In panels **(G–I)**, genomic DNA of *F. pratensis* was labeled with digoxigenin (green coloring), while genomic DNA of *L. multiflorum* served as blocking DNA and all chromosomes were counterstained with DAPI (red pseudocolor).

The *Ph1* locus is present in tetraploid wheat plants as well, such as *T. turgidum* subsp. *durum* (Dvorak et al., 1984) and *T. timopheevi* subsp. *timopheevi* (Feldman, 1966b). In the latter, a mutant for this particular gene was developed, called *ph1c*, having a similar phenotype as the hexaploid mutant *ph1b*, i.e., increased homoeologous chromosome chiasmata in metaphase I (Jauhar et al., 1999). In a comparative study assessing the effectiveness of *Ph1* gene in tetraploid and hexaploid wheat, Ozkan and Feldman (2001) crossed *Ae. peregrina* with hexaploid wheat and derivative lines, wherein chromosome 5B was replaced by its variant from tetraploid wheat (either from *T. turgidum* subsp. *dicoccoides* or *T. timopheevi* subsp. *Timopheevi*). With 5B from tetraploid wheat present, a higher frequency of homoeologous chromosome associations was observed in hybrids relative to the presence of endogenous 5B, indicating the tetraploid variant of *Ph1* gene might operate with lower effectiveness. Interestingly, once *Ph1* is introgressed from wheat into related species, its ability to modify chromosome bahavior is also preserved in the host genome (**Figures 2A, B**; Lukaszewski and Kopecký, 2010).

The *Ph1* regulator probably acts in multiple ways during meiosis. In early prophase I, it promotes the formation and subsequent correction of synapses (Holm, 1986; Martínez et al., 2001a), but later on, it affects the frequency of cross-over formation (Martín et al., 2014). Originally, the *Ph1* gene was thought to function as a suppressor of homoeologous synapses (Holm and Wang, 1988), but the current view is that it works primarily by promoting and stabilizing homologous synapses (Martín et al., 2017). During metaphase I in hexaploid wheat, ring bivalents are predominantly formed between homologous chromosomes, with some rod bivalents occurring in all meiocytes (Martín et al., 2014). In the *ph1b* mutant, only ~50% of meiocytes wil display similar meiotic behavior with increased frequency of rod bivalents; in the other half, variable numbers of multivalents and univalents were instead detected. This means that roughly half of the meiocytes display chiasmata only between homologous chromosomes (Martín et al., 2014). Similarly, other studies could not find homoeologous chiasmata in significant fractions of meiocytes in other *Ph1* mutants (Roberts et al., 1999; Al-Kaff et al., 2008; King et al., 2016). This suggests the promotion of homologous synapses is the main function of the *Ph1* gene, rather than suppression of homoeologous ones (Martín et al., 2017). This hypothesis is further supported by the higher occurrence of univalents in *ph1b* mutants than in the wild type or *ph2b* mutant (**Table 1**).

Griffiths et al. (2006) performed a screen for a *ph1*-like phenotype in the population of EMS mutants. Yet they failed to find an individual showing the full *ph1b*-like phenotype. This indicates the *Ph1* phenotype might not be under the control of a single gene. The *Ph1* locus was further narrowed down to a 2.5-Mb region on the long arm of the 5B chromosome (Griffiths et al., 2006), which contains a duplicated segment from chromosome 3B composed of a cluster of *Cdk2-*like kinases and methyl-transferase genes (Griffiths et al., 2006; Al-Kaff et al., 2008; Martín et al., 2017). The Cdk-like kinases in the locus show close homology to the mammalian Cdk2, which is essential for homologous chromosome recognition and recombination (Ortega et al., 2003; Viera et al., 2009). Two groups of researchers disagree on which of the genes located in this particular region is the one responsible for promotion of homologous chiasmata. Bhullar et al. (2014) proposed *C-Ph1* (RAFTIN1-like protein containing BURP domain) to be a putative *Ph1* gene, but deletion lines for *C-Ph1* locus failed to produce the same phenotype as the *ph1b* mutant (Al-Kaff et al., 2008). Moreover, the rice homolog and wheat paralog of this gene were already shown to be specific to tapetal cells (Jeon et al., 1999; Wang et al., 2003). The other group proposed a different candidate, a paralog of *ZIP4*. The encoded protein affects the homologous cross-overs in *Arabidopsis* and rice, supporting the assumption that this gene could be responsible for the *Ph1* phenotype (Chelysheva et al., 2007; Shen et al., 2012; Rey et al., 2017). Both EMS and CRISPR mutations for this gene (named *TaZIP4-B2)* promoted homoeologous cross-overs in hybrids between wheat and *Ae. variabilis* (Rey et al., 2017; Rey et al., 2018). But these hybrids did not show the same extent of multivalent formation or an increase in univalents as typically observed in hybrids between the *ph1b* mutant and *Ae. variabilis*. Nevertheless, these results do suggest the *TaZIP4-B2* plays an important role in the control of homoeologous pairing in wheat (Rey et al., 2017; Rey et al., 2018; Naranjo, 2019). The putative additional effector in this region has yet to be identified.

#### *Pairing Homoeologous 2 (Ph2)*

Another gene, called *Ph2*, has a weaker effect (than *Ph1*) on homologous chromosome pairing in wheat. That gene was assigned to chromosome 3D by Mello-Sampayo (1968; 1971) who observed multivalent formation in metaphase I in the absence of chromosome 3D in pentaploid hybrids between *T. aestivum* and *T. durum*, as well as in hybrids between *T. aestivum* and *Aegilops*. Two *Ph2* mutants were since developed; the X-ray-induced mutant *ph2a* carrying a large deletion (Sears, 1982), and the chemically-induced (EMS) mutant *ph2b* (Wall et al., 1971). Using both mutants, the *Ph2* phenotype was studied and the locus narrowed down, using synteny with rice, to a terminal 80 Mb of the short arm of chromosome 3D (Sutton et al., 2003). More recently, however, Svačina et al. (2020) showed that this deletion in the *ph2a* mutant is actually larger than expected, comprising about 125 Mb terminal part of the short arm of chromosome 3D.

The *Ph2* gene operates in a different way than does *Ph1* (Benavente et al., 1998; Martinez et al., 2001a). Both Martinez et al., (2001a) and Sánchez-Morán et al. (2001) evaluated the effect of its mutations in hexaploid wheat, finding no visible influence upon homoeologous chiasmata when *Ph1* is present and *Ph2* absent, apart from a slight increase in univalent formations. Earlier, Sears (1977; 1982) had shown that in hybrids of wheat and closely related species, moderate frequency of homoeologous chiasmata happened in the absence of *Ph2* but in the presence of *Ph1*. In the case of wheat-rye hybrids lacking the *Ph2* locus, Prieto et al. (2005) also observed an intermediate number of homoeologous chiasmata; however, according to their GISH analysis, the chromosome associations only occur between wheat chromosomes, whereas wheat–rye associations were rare similarly to the wild-type hybrid. This contrasts with the *ph1b* mutant, for which some frequency of wheat–rye associations was detectable (refer to **Table 2**; Prieto et al., 2005). These findings suggest to us that *Ph2* plays a diminished functional role when homologous chromosomes are present (**Table 1**). Yet, in the absence of homologs, it may well suppress associations among homoeologous chromosomes. Furthermore, researchers discovered that *Ph2* has a different function to that of *Ph1* as it is not involved in recognition of homologous chromosomes but instead affects the progression of synapsis (Martinez et al., 2001a; Prieto et al., 2005). We should also not overlook possible cooperation between *Ph1* and *Ph2* in their modes of action, as suggested by the work of Boden et al. (2009).

**Table 2:** Number of chromosome-arm associations in metaphase I in haploid hybrids derived from the crossing of rye with euploid wheat (CS, ‘Chinese Spring’) and *ph1b* and *ph2b* mutants (Prieto et al., 2005).

Genotype	CS × rye	*ph2b* × rye	*ph1b* × rye
Chromosome number	28	28	28
Wheat–wheat	0.48	1.68	7.14
Wheat–rye	0.08	0.08	0.59
Rye–rye	0.02	0.04	0.05
Total	0.58	1.80	7.78

The *ph2a* mutant has been exploited in trying to identify candidate genes underlying its phenotype. Many have been proposed, such as *TaMSH7*, the homolog of the *MSH6* DNA mismatch repair gene in yeast (Dong et al., 2002), in addition to the *WM5* (Thomas, 1997) and *WM1* gene family members (Ji and Langridge, 1994; Whitford, 2002). Sutton et al. (2003) used comparative genetics to further identify the putative genes involved in the *Ph2* phenotype; however, no clear candidate producing a mutant phenotype similar to the *ph2a* has been identified.

#### Meiotic Behavior in Hybrids of *ph* Mutants and Wild-Type Wheat With Closely Related Species

The pairing of homoeologous chromosomes is mostly studied in haploids or interspecific hybrids, that is, in the absence of homologous chromosomes, the natural partners for pairing. The exent of chromosome associations during metaphase I of meiosis, in hybrids of wild-type hexaploid wheat or *ph2b* and *ph1b* mutants with various relatives, will differ based on the degree of homology between the genomes involved. The frequency of homoeologous chromosome chiasmata increases when there is a closer phylogenetic relationship of the parents. The fewest homoeologous associations were observed in the hybrids between hexaploid wheat and rye (**Table 3**; Naranjo et al., 1987; Naranjo et al., 1988). This can be explained by the fact that lineages towards wheat and rye split about 7 MYA while *Aegilops* diverged from wheat 2.5–5.0 MYA (Huang et al., 2002). Accordingly, the *Aegilops* chromosomes are more closely related to wheat chromosomes than those of rye. The highest frequency of homoeologous chromosome associations was observed in the hybrid of hexaploid wheat and *Ae. speltoides* (Maestra and Naranjo, 1998; **Table 3**); the latter is a species closely related to the donor of the B genome in wheat, and thus highly similar to one of the wheat genomes (Huang et al., 2002; Petersen et al., 2006). These observations suggest the *Ph* system’s recognition of homologous chromosomes begins to fail with increasing homology between genomes in the hybrid, resulting in homoeologous chromosome chiasmata. Alternatively, there may exist genes that suppress or interfere with the *Ph* system in certain species used for hybridization with wheat (see below).

**Table 3:** Associations of homoeologous chromosomes in metaphase I in various hybrids of wild-type wheat (WT) and *ph1b* and *ph2b* mutants with closely related plant species (Naranjo et al., 1987; Naranjo et al., 1988; Naranjo and Maestra, 1995; Maestra and Naranjo, 1997; Maestra and Naranjo, 1998).

Hybrid	Chromosome number	Univalents	Rod bivalents	Ring bivalents	Multivalents	Chiasmata percell
WT × rye	28	26.31	0.80	0.03	0.01	0.88
*ph2b* × rye	28	19.23	3.4	0.57	0.51	5.26
*ph1b* × rye	28	11.76	2.33	2.36	2.16	12.35
WT × *Ae. longissima*	28	24.55	1.59	0.06	0.05	1.81
*ph2b* × *Ae. longissima*	28	14.93	5.8	0.58	0.55	7.44
*ph1b* × *Ae. longissima*	28	3.48	4.4	2.99	2.86	18.28
WT × *Ae. sharonensis*	28	25.21	1.18	0.03	0.03	1.29
*ph2b* × *Ae. sharonensis*	28	10.16	5.58	1.42	1.13	11.17
*ph1b* × *Ae. sharonensis*	28	4.37	3.74	3.79	2.39	17.93
WT × *Ae. speltoides*	28	3.97	4.9	3.11	2.61	17.79
*ph2b* × *Ae. speltoides*	28	3.25	3.41	3.28	3.2	19.41
*ph1b* × *Ae. speltoides*	28	2.53	3.36	4.29	2.68	20.08

#### Homoeologous Chromosome Associations in the Presence of *Ph* Genes

*Ph* genes ensure that only homologous chromosome chiasmata occur in polyploid wheat during meiosis. However, the functioning of these genes can be suppressed in some hybrids, resulting in increased homoeologous chromosome associations; e.g., in hybrids of *T. aestivum* with *Ae. speltoides* or *Ae. mutica* (Riley, 1960; Dover and Riley, 1972; Dvorak et al., 2006a). For the wheat *×* *Ae. speltoides* hybrid, Dvorak et al. (2006b) identified two suppressors on chromosomes 3S (*Su1-Ph1*) and 7S (*Su2-Ph1*) that affected homoeologous chromosome associations, varying from 7.0 to 16.4 chiasmata per cell. The *Su1-Ph1* was introgressed into both hexaploid and tetraploid wheat, opening new possibilities in inducing homoeologous chromosome recombinations for introgression into wheat (Li et al., 2017). This phenomenon can also be observed in lines where only a single chromosome was introgressed into the wheat background. In particular, the presence in wheat of chromosome 5U from *Ae. umbellulata* (Riley et al., 1973), or that of chromosome 5E from *Elytrigia elongata* (Dvorak, 1987), promotes homoeologous chromosome chiasmata with the formation of trivalents and bivalents in the haploids (ABD + 5U; ABD + 5E). This outcome suggests that introducing some alien chromosomes can suppress the functioning of *Ph* genes (Koo et al., 2017). Another case of homoeologous chromosome associations in the presence of *Ph* genes was reported on by Liu et al. (2011), who observed frequent recombination between 5M^g^ and 5D chromosomes in substitution lines containing 5M^g^ from *Ae. geniculata*. Later, Koo et al. (2017) used two different 5M^g^ chromosomes from different accessions in the wheat background and observed differential associations between 5M^g^ and 5D in both lines, for which chiasmata between 5M^g^ and 5D were detected in 6.7% and 21.7% of ensuing meiocytes. This might have been caused by the presence of genes located on the particular alien chromosome either actively promoting homoeologous chromosome chiasmata or repressing *Ph1*. Additionally, homoeologous associations probably occurred only between the 5M^g^ and 5D chromosome, as no multivalent was detected (Koo et al., 2017). In another example, homoeologous barley chromosomes fully associated in pairs in the presence of *Ph1* (Martín et al., 2017; Calderón et al., 2018). However, these homoeologous chromosomes did not cross-over, suggesting that *Ph1* does not prevent chromosome pairing between homoeologs, but supresses its recombination (Calderón et al., 2018).

In a natural population of the Chinese landrace of hexaploid wheat ‘Kaixianluohanmai’ (KL), another gene promoting homoeologous chiasmata in wheat–alien hybrids (presumably in presence of *Ph*) was posited (Luo et al., 1992). Meiosis is regular and normal in KL wheat by itself, as in other wheat landraces (Fan et al., 2019), but a moderate frequency of homoeologous chromosome associations occurs in hybrids of KL wheat with rye and *Aegilops variabilis* (similar as that between *ph1b ×* rye and *ph2b ×* rye hybrids) (**Table 4**; Luo et al., 1992; Liu et al., 1998; Liu et al., 2003; Xiang et al., 2005). In hybrids arising between KL wheat and *Psathyrostachys huashanica*, the frequency of homoeologous chromosome chiasmata even exceeded that of the *ph1b* *×* *P. huashanica* hybrid (Kang et al., 2008). This locus, named *phKL*, is most probably not allelic to either *Ph1* or *Ph2* (Liu et al., 2003; Hao et al., 2011). The analysis of monosomics did show that a locus on chromosome 6A in KL might be responsible for the *phKL* phenotype (Liu et al., 1997). However, using two mapping populations, Fan et al. (2019) recently identified a QTL locus possibly responsible for homoeologous associations on chromosome arm 3AL.

**Table 4:** Chromosome associations in metaphase I in hybrids derived from crossings of rye with the wheat KL landrace, “Chinese Spring” (CS), and the Chinese Spring *ph1* (*CSph1b*) and *ph2* (*CSph2a*) mutants (Hao et al., 2011).

Genotype	Number of associations per cell			
	Rod	Ring	Multivalent	Chiasmata
KL × rye	4.73	0.20	0.11	5.40
CS*ph1b* × rye	4.85	1.87	0.47	9.53
CS*ph2a* × rye	1.74	0.00	0.02	1.78
CS × rye	0.54	0.00	0.00	0.54

### Chromosome-Pairing Regulators in Other Poaceae Taxa

Bread wheat is undoubtedly the most studied and well-understood species concerning the mechanism of homologous chromosome recognition in the Poaceae family. Nonetheless, clues to the presence of similar machinery has been observed in other grass species, namely in *Avena* spp. (Ladizinsky, 1973), *Oryza* spp. (Cai et al., 2004), *Festuca* spp. (Jauhar, 1993), polyploid *Hordeum* spp. (Gupta and Fedak, 1985), or *Alopecurus* spp. (Murray et al., 1984). Several examples of chromosome associations in allo- and autopolyploids from the Poaceae family are shown in **Figure 2**.

The genus *Festuca* comprises over 500 species having a wide range of ploidy levels, from diploids to dodecaploids (Loureiro et al., 2007). Agriculturally most important are those species from the subgenus *Schedonorus* comprising broad-leaved fescues, the majority of which are polyploids, from tetraploids to decaploids (Kopecký et al., 2008b). Molecular and cytogenetic analyses have revealed that all these studied polyploid species arose from interspecific hybridization (Humphreys et al., 1995; Catalán and Olmstead, 2000; Hand et al., 2010; Ezquerro-López et al., 2017); hence, they are of allopolyploid origin. All these allopolyploid species—including the tetraploids *F. mairei*, *F. apennina*, and *F. glaucescens*, hexaploid *F. arundinacea*, and octoploids *F. arundinacea* subsp. *atlantigena* and decaploid *F. arundinacea* var. *letourneuxiana*—possess diploid-like pairing behavior during meiosis, with bivalent formation (reviewed in Jauhar, 1993). Jauhar (1975) had proposed the existence of a homoeologous-pairing suppressor in tall fescue (*F. arundinacea*, 2n = 6*x* = 42; FpFpFgFgFg’Fg’) (**Figure 2C**). He found frequent multivalent formations in haploid plants of tall fescue (2n = 3*x* = 21) and speculated on the haplo-insufficiency or hemizygous-ineffectivity of the system: meaning that two copies of such gene(s) must be present for the induction of strict homologous pairing. This differentiates the fescues’ system from *Ph1* of wheat and the regulator found in oats (Jauhar, 1993). Another difference is that *Ph1* can supress homeologous recombination and/or promote homologous ones, while the control system in tall fescue seems to be responsible for the formation of homologous bivalents. Colchicine-induced dodecaploid wheat was able to form quadrivalents composed of four homologous chromosomes, whereas only homologous bivalents formed in the synthetically derived dodecaploid tall fescue plant (Jauhar, 1975).

Where the gene(s) underpinning diploid-like pairing system is located on one or more particular chromosomes or even subgenomes of tall fescue plants remains unknown. In tetraploid tall fescue (FpFpFgFg’), homoeologous chromosomes form chiasmata frequently; moreover, the frequent formation of quadrivalents was recorded in colchicine-induced autotetraploids of *F. pratensis* (**Figure 2E**; Kopecký et al., 2009). Thus, one of the subgenomes originating from *F. glaucescens* must harbor the responsible gene(s) (**Figure 2D**). In early work, Jauhar (1975) analyzed a set of monosomic lines of tall fescue and found one line with disrupted diploid-like behavior, probably due to an absence of the chromosome carrying the gene(s) for diploid-like pairing behavior. Unfortunately, this line was lost over time and so it cannot be further investigated. Later, Kleijer and Morel (1984) speculated that disruption of strictly homologous associations in a single plant is more likely to be only a consequence of normal variation among plants. The system may also interfere with other systems present in the genus, or in closely related genera. A high frequency of quadrivalents was observed in the tetraploid *Lolium multiflorum* *×* *F. arundinacea* hybrid (LmFpFgFg’) (**Figure 2F**), which exceeded that of quadrivalents in tetraploid *F. arundinacea* (FpFpFgFg’) (Kopecký et al., 2009).

The origin of the system in polyploid fescues is not known, but several scenarios are plausible. It could have developed in a currently unknown diploid species, which served as a progenitor of all recent polyploid species. Alternatively, such a system arose in an early-day polyploid (presumably an allotetraploid), since involved in the evolution of other allopolyploids. Support for both scenarios lies in the fact that the system in all species has the same (rare) attribute: haplo-insufficiency. The third possible scenario involves multiple origins of the system in different species during their evolutionary history. Or, the system is the outcome of two scenarios combined. It does seem that the systems found in various species are compatible in some hybrid combinations yet dysfunctional in others. Eizenga et al. (1990) found that multivalents were rare in the hybrids of tall fescue and giant fescue (*F. gigantea*). Similarly, hybrids of *F. mairei* × *F. glaucescens* show preferential formation of bivalents with a very low frequency of multivalents (nine quadrivalents and one trivalent among 200 PMCs [pollen mother cells]) (Malik and Thomas, 1967). By contrast, the hybrids of Continental and Mediterranean morphotypes of tall fescue all display high levels of multivalent formation (Kopecký et al., 2019), suggesting incompatibility of the two regulatory systems, or some epistatic effects. Therefore, we cannot unambiguously clarify if the system evolved once or twice (or even more times). However, if it did develop just once, the system diverged in different species during evolution to reach a level of incompatibility, as evinced from the analyses of interspecific hybrids.

The genus oat (*Avena* spp.) consists of diploid, tetraploid, and hexaploid species, including the important crop *A. sativa*. Polyploid oats include both auto- and allopolyploid forms, whose diploid-like behavior in meiosis is preserved despite partial homology between their genomes, suggesting the existence of a *Ph*-like system (Thomas, 1992). Oats comprise four cytologically distinct genomes (A, B, C, and D), however the genomes B and D occur only in polyploid taxa (Leggett and Thomas, 1995). Similar to wheat, the system found in tetraploid and hexaploid oats is hemizygous effective and haplo-sufficient, and susceptible to dosage effects and genetic repressibility. The locus that contains the gene(s) for meiotic regulation is likely localized to the A genome (Jauhar, 1977). Unfortunately, surpisingly little is known about the genes whose activity maintains homologous chromosome pairing in oats, apart from their existence being proven by increased associations among homoeologous chromosomes in some nulli-haploid *A. sativa* lines (Gauthier and McGuinnis, 1968).

## Polyploidy and Homoeologous Chromosome Pairing in Plant Breeding

Besides its key role in plant speciation, polyploidization and hybridization are popular tools in plant breeding. The most straightforward agronomical effect of polyploidy is an increased cell size, potentially resulting in larger organs, including fruits, roots, flowers, leaves, and seeds (Stebbins, 1950). Another frequent consequence of polyploidy is sterility, which generally has an agronomically negative effect; however, for seedless fruit production it can be a desirable trait, as in triploid seedless watermelon (Crow, 1994). The fixation of heterozygosity in allopolyploid species often leads to heterosis, resulting in higher vigor of the hybrids compared with their diploid progenitors, such as in hexaploid wheat *T. aestivum* (Sattler et al., 2016). Wide hybridization coupled to whole genome duplication is commonly used to merge beneficial inheritable traits from both parents, namely in the introgression of a chromosome segment carrying genes for a desirable trait from the wild relative to elite crop cultivars, or for simply widening the gene pool. One of the most promising artificially developed hybrids is Triticale, which originated from the crossing of wheat and rye with a subsequent chromosome doubling (Meister and Tjumjakoff, 1928).

One of the key components for the successful utilization of wide hybridization in plant breeding is the control of homoeologous chromosome associations. In countless studies, the *ph1b* mutant of wheat has been used to induce homoeologous chromosome recombinations between chromosomes of wheat and related species, for transferring desirable traits into the wheat genome (Marais et al., 2010; Niu et al., 2011; Ayala-Navarrete et al., 2013; Rey et al., 2015a; Rey et al., 2015b; Han et al., 2016; King et al., 2019). After the introgression of the chromosomal segment from a related species, it is necessary to immediately re-activate the *Ph1* gene to avoid risking the rapid elimination of the segment. Nevertheless, some hybrids without meiotic regulation but with homoeologous chromosome pairing can be valuable also and remain relatively stable. Complementary attributes of ryegrasses (i.e., high yield and nutrition) and fescues (i.e., abiotic stress tolerance) can be combined in their hybrids called Festulolium. In last 50 years, many agriculturally successful cultivars have been released *via* several breeding programs (Ghesquière et al., 2010). To do this, the breeders often used tetraploid parents for the initial mating. Such F1 Festulolium hybrids are all allotetraploids and possess two sets of chromosomes from both parental species. One would presume that homologous chromosome associations would be the predominant mode of action due to variation in the DNA sequence. The repetitive elements from these two genera diverged sufficiently that it is now possible to distinguish chromosomes of *Festuca* from those of *Lolium* by genomic *in situ* hybridization (GISH) (Thomas et al., 1994). Yet, frequent formation of homoeologous chromosome chiasmata has been detected in F1 hybrids, as well as in monosomic and disomic substitution lines of *L. multiflorum* × *F. pratensis* (**Figures 2G, H**; Kopecký et al., 2008a). Such massive homoeologous associations and recombination leads to highly variable karyotypes differing from plant to plant (**Figure 2I**). An outcrossing mode of reproduction augments this variability within each population of hybrids over subsequent generations. Consequently, both high variability and heterosis ensue within the bred plant material. It is nevertheless possible to uniform the breeding material at a phenotypic level to the extent that it passes DUS tests for registration as a commercial cultivar. While the proportion of parental genomes was relatively stable in subsequent generations of three commercial hybrids (Kopecký et al., 2008a), substantial variability was found within populations of each generation of those cultivars.

Besides those amphiploid (or allotetraploid) cultivars, introgression breeding may also be used to develop Festulolium cultivars. Doing this involves at least one round of backcrossing of F1 hybrids with one of the parental species (usually *Lolium*), giving rise to plants similar to the parental species but with improved characteristics, such as frost tolerance or higher survivorship (reviewed in Kopecký et al., 2008b). Karyologically, these plants usually carry only one or few chromosome segments of *Festuca*. Such introgression lines are usually highly unstable and the introgressed segment(s) is/are often lost in subsequent generations (Kopecký et al., 2019). Accordingly, implementing any system capable of preventing associations of homoeologous chromosomes is arguably desirable to stabilize the genomic composition of hybrids. In amphiploids, immediate introgression of the system would be required to keep both parental subgenomes intact. To date, most cultivars have originated from the cross of *L. multiflorum* × *F. pratensis*, though none of the parents carry a homoeologous suppressor. Instead, tetraploid wild relatives, such as *F. glaucescens*, *F. apennina* and *F. mairei*, which possess a meiotic regulator hampering homoeologous pairing, should be considered for future crosses as they are known for their tolerance to biotic and abiotic stress, which might complement the high yield and nutrition traits of ryegrasses. In this respects, first attempts have been made and the cultivar of *L. multiflorum* × *F. glaucescens ‘Lueur’* was registered in France (Ghesquière et al., 2010) and other similar cross combinations are used in breeding programs in both the UK and Czech Republic. Considering the haplo-insufficiency of the system found in polyploid fescues, evidently the F1 hybrids will possess some level of homoeologous associations. Still, it should be possible to select F2 plants that have two copies of the gene(s) of the system and then intercross them. Doing this should facilitate the stabilization of the hybrid genome in successive generations. For the corresponding introgression lines, the segment carrying the gene(s) of the system must be present among the introgressions. Thereafter, haploidization, followed by either spontaneous or induced chromosome doubling, should result in the establishment of plants having two copies of such gene(s) required for its/their functionality as the homoeologous pairing suppressor(s). Clearly, though, further investigation of chromosome behavior in fescues is necessary if we hope to foster genetically stable grass hybrids.

We envisage that with more knowledge of the mechanisms responsible for correct chromosome associations, the efficient employment of targeted interspecific hybridization techniques will become available in the near future. Perhaps the most challenging task is the developing and operating of an “OFF” and “ON” switch to control recombination of homoeologous chromosomes. It would be immensely helpful for breeders to switch “OFF” the system in wheat and other allopolyploids with an established and functional regulatory system for introgressing the specific segment from a wild relative. Once the segment is transferred, the switch to “ON” would then stabilize the segment and permit its proper transmission into successive generations. Similarly, introgression of the system into a hybrid (originally lacking the regulator) with desirable combinations of parental chromatin would assist in further stabilizing the hybrid genome composition. To conclude, additional research broadening our knowledge of the mechanisms governing meiotic chromosome behavior in allopolyploids is necessary to ensure further success in future breeding of grass plants.

## Author Contributions

RS, PS, DK, and JB wrote the manuscript.

## Funding

This work was supported by the Czech Science Foundation (grant award 17-05341S) and the ERDF project “Plants as a tool for sustainable global development” (CZ.02.1.01/0.0/0.0/16_019/0000827).

## Conflict of Interest

The authors declare that the research was conducted in the absence of any commercial or financial relationships that could be construed as a potential conflict of interest.

## References

[B1] Akhunov E. D. Sehgal S. Liang H. Wang S. Akhunova A. R. Kaur G. (2013). Comparative analysis of syntenic genes in grass genomes reveals accelerated rates of gene structure and coding sequence evolution in polyploid wheat. Plant Physiol. 161, 252–265. 10.1104/pp.112.205161 23124323 PMC3532256

[B2] Al-Kaff N. Knight E. Bertin I. Foote T. Hart N. Griffiths S. (2008). Detailed dissection of the chromosomal region containing the ph1 locus in wheat *Triticum aestivum*: with deletion mutants and expression profiling. Ann. Bot. 101, 863–872. 10.1093/aob/mcm252 17951583 PMC2710213

[B3] Aragón-Alcaide L. Reader S. Beven A. Shaw P. Miller T. Moore G. (1997). Association of homologous chromosomes during floral development. Curr. Biol. 7, 905–908. 10.1016/S0960-9822(06)00383-6 9382806

[B4] Attia T. Ekingen H. Röbbelen G. (1979). Origin of 3D-suppressor of homoeologous pairing in hexaploid wheat. Z. Pflanzenzüchtg 83, 121–126.

[B5] Aung T. Evans G. M. (1985). The potential for diploidizing *Lolium multiflorum* × *L.* *perenne* tetraploids. Can. J. Genet. Cytol. 27, 506–509. 10.1139/g85-075

[B6] Ayala-Navarrete L. II Mechanicos A. A. Gibson J. M. Singh D. Bariana H. S. Fletcher J. (2013). The Pontin series of recombinant alien translocations in bread wheat: single translocations integrating combinations of Bdv2, Lr19 and Sr25 disease-resistance genes from *Thinopyrum intermedium* and *Th.* *ponticum* . Theor. Appl. Genet. 126, 2467–2475. 10.1007/s00122-013-2147-0 23807636

[B7] Balfourier F. Bouchet S. Robert S. DeOliveira R. Rimbert H. Kitt J. (2019). Worldwide phylogeography and history of wheat genetic diversity. Sci. Adv. 5 (5), eaav0536. 10.1126/sciadv.aav0536 31149630 PMC6541461

[B8] Barker M. S. Arrigo N. Baniaga A. E. Li Z. Levin D. A. (2016). On the relative abundance of autopolyploids and allopolyploids. New Phytol. 210, 391–398. 10.1111/nph.13698 26439879

[B9] BassH. W.Riera-LizarazuO.AnanievE. V.BordoloS. J.RinesH. W.PhillipsR. L. (2000). Evidence for the coincident initiation of homologue pairing and synapsis during the telomere clustering (bouquet) stage of meiotic prophase. J. Cell Sci. 113, 1033–1042.1068315110.1242/jcs.113.6.1033

[B10] Bass H. W. (2003). Telomere dynamics unique to meiotic prophase: formation and significance of the bouquet. Cell. Mol. Life Sci. 60, 2319–2324. 10.1007/s00018-003-3312-4 14625678 PMC11138934

[B11] Baumel A. Ainouche M. L. Levasseur J. E. (2001). Molecular investigations in populations of *Spartina anglica* C.E. Hubbard (Poaceae) invading coastal Brittany (France). Mol. Ecol. 10, 1689–1701. 10.1046/j.1365-294X.2001.01299.x 11472537

[B12] Benavente E. Orellana J. Fernández-Calvín B. (1998). Comparative analysis of the meiotic effects of wheat *ph1b* and *ph2b* mutations in wheat×rye hybrids. Theor. Appl. Genet. 96, 1200–1204. 10.1007/s001220050857

[B13] Bhullar R. Nagarajan R. Bennypaul H. Sidhu G. K. Sidhu G. Rustgi S. (2014). Silencing of a metaphase I-specific gene results in a phenotype similar to that of the pairing homeologous 1 (Ph1) gene mutations. Proc. Natl. Acad. Sci. U.S.A. 111, 14187–14192. 10.1073/pnas.1416241111 25232038 PMC4191769

[B14] Boden S. A. Langridge P. Spangenberg G. Able J. A. (2009). TaASY1 promotes homologous chromosome interactions and is affected by deletion of Ph1. Plant J. 57, 487–497. 10.1111/j.1365-313X.2008.03701.x 18826431

[B15] Brown W. V. Stack S. M. (1968). Somatic pairing as a regular preliminary to meiosis. Bull. Torrey Bot. Club 95, 369–378. 10.2307/2483872

[B16] Cai D. T. Chen D. L. Chen J. G. Liu Y. Q. (2004). A method of induction polyploidy rice with high frequency through tissue culture together with chemical agent induction. China Patent: ZL01133529.7.

[B17] Cai D. T. Chen J. Chen D. Dai B. C. Song Z. J. Yang Z. F. (2007). The breeding of two polyploid rice lines with the characteristic of polyploid meiosis stability. Sci. China Ser. C. 50, 356–366. 10.1007/s11427-007-0049-6 17609893

[B18] Calderón M. C. Rey M. D. Martín A. Prieto P. (2018). Homoeologous Chromosomes From Two Hordeum Species Can Recognize and Associate During Meiosis in Wheat in the Presence of the *Ph1* Locus. Front. Plant Sci. 9, 585. 10.3389/fpls.2018.00585 29765389 PMC5938817

[B19] Carvalho A. Delgado M. Barão A. Frescatada M. Ribeiro E. Pikaard C. S. (2010). Chromosome and DNA methylation dynamics during meiosis in the autotetraploid *Arabidopsis arenosa* . Sex Plant Reprod. 23, 29–37. 10.1007/s00497-009-0115-2 20165961

[B20] Catalán P. Olmstead R. G. (2000). Phylogenetic reconstruction of the genus *Brachypodium* P. Beauv. (*Poaceae*) from combined sequences of chloroplast *ndhF* gene and nuclear ITS. Pl. Syst. Evol. 220, 1–19. 10.1007/BF00985367

[B21] Chelysheva L. Gendrot G. Vezon D. Doutriaux M. P. Mercier R. Grelon M. (2007). *Zip4*/*Spo22* is required for class I CO formation but not for synapsis completion in *Arabidopsis thaliana* . PloS Genet. 3, e83. 10.1371/journal.pgen.0030083 17530928 PMC1877879

[B22] Clausen J. Keck D. D. Hiesey W. M. (1945). Experimental studies on the nature of species, II. Plant evolution through amphiploidy and autopolyploidy, with examples from the Madiinae (Washington, DC.: Carnegie Institute of Washington).

[B23] Comai L. (2005). The advantages and disadvantages of being polyploid. Nat. Rev. Genet. 6, 836–846. 10.1038/nrg1711 16304599

[B24] Comings D. E. (1968). The rational for an ordered arrangement of chromatin in the prophase nucleus. Am. J. Hum. Genet. 20, 440–460. 5701616 PMC1706348

[B25] Crow J. F. (1994). Hitoshi Kihara, Japan’s pioneer geneticist. Genetics 137, 891–894. 7982570 10.1093/genetics/137.4.891 PMC1206066

[B26] Dawe R. K. (1998). Meiotic chromosome organization and segregation in plants. Ann. Rev. Plant Physiol. Plant Mol. Biol. 49, 371–395. 10.1146/annurev.arplant.49.1.371 15012239

[B27] De Storme N. Copenhaver G. P. Geelen D. (2012). Production of diploid male gametes in *Arabidopsis* by cold-induced destabilization of postmeiotic radial microtubule arrays. Plant Physiol. 160, 1808–1826. 10.1104/pp.112.208611 23096158 PMC3510112

[B28] Dong C. Whitford R. Langridge P. (2002). A DNA mismatch repair gene links to the *Ph2* locus in wheat. Genome 45, 116–124. 10.1139/g01-126 11908653

[B29] Dover G. A. Riley R. (1972). Prevention of pairing of homoeologous meiotic chromosomes of wheat by an activity of supernumerary chromosomes of *Aegilops* . Nature 240, 159–161. 10.1038/240159a0

[B30] Driscoll C. J. (1972). Genetic suppression of homoeologous chromosome pairing in hexaploid wheat. Can. J. Genet. Cytol. 14 (1), 39–42. 10.1139/g72-004

[B31] Dubcovsky J. Dvorak J. (2007). Genome plasticity a key factor in the success of polyploid wheat under domestication. Science 316, 1862–1866. 10.1126/science.1143986 17600208 PMC4737438

[B32] Dvorak J. Chen K. C. Giorgi B. (1984). The C-banding pattern of a Ph-mutant of durum wheat. Can. J. Genet. Cytol. 26, 360–363. 10.1139/g84-056

[B33] Dvorak J. Akhunov E. D. Akhunov A. R. Deal K. R. Luo M. C. (2006a). Molecular characterization of a diagnostic DNA marker for domesticated tetraploid wheat provides evidence for gene flow from wild tetraploid wheat to hexaploid wheat. Mol. Biol. Evol. 23, 1386–1396. 10.1093/molbev/msl004 16675504

[B34] Dvorak J. Deal K. R. Luo M. C. (2006b). Discovery and mapping of wheat Ph1 suppressors. Genetics 174, 17–27. 10.1534/genetics.106.058115 16702426 PMC1569802

[B35] Dvorak J. (1987). Chromosomal distribution of genes in diploid *Elytrigia elongata* that promote or suppress pairing of wheat homoeologous chromosomes. Genome 29, 34–40. 10.1139/g87-006

[B36] Eber F. Chèvre A. M. Baranger A. Vallée P. Tanguy X. Renard M. (1994). Spontaneous hybridization between a male-sterile oilseed rape and two weeds. Theor. Appl. Genet. 88, 362–368. 10.1007/BF00223646 24186020

[B37] Ehrendorfer F. (1980). ““Polyploidy and Distribution,”,” in Polyploidy. Basic Life Sciences, vol. 13 . Ed. Lewis W. H. (Boston, MA: Springer), 45–60. 10.1007/978-1-4613-3069-1_3 233065

[B38] Eilam T. Anikster Y. Millet E. Manisterski J. Feldman M. (2009). Genome size in natural and synthetic autopolyploids and in a natural segmental allopolyploid of several *Triticeae* species. Genome 52, 275–285. 10.1139/G09-004 19234556

[B39] Eizenga G. C. Burner D. M. Buckner R. C. (1990). Meiotic and isozymic analyses of tall fescue × giant fescue hybrids and amphiploids. Plant Breed. 104, 202–211. 10.1111/j.1439-0523.1990.tb00424.x

[B40] Evans G. M. Aung T. (1985). Identification of a diploidizing genotype of *Lolium multiflorum* . Can. J. Genet. Cytol. 27, 498–505. 10.1139/g85-074

[B41] Evans G. M. Aung T. (1986). The influence of the genotype of *Lolium perenne* on homoeologous chromosome association in hexaploid *Festuca arundinacea* . Heredity 56, 97–103. 10.1038/hdy.1986.13

[B42] Evans G. M. Macefield A. J. (1972). Suppression of homoeologous pairing by B chromosomes in a *Lolium* species hybrid. Nat. New Biol. 236, 110–111. 10.1038/newbio236110a0 4502805

[B43] Evans G. M. Macefield A. J. (1973). The effect of B chromosomes on homoeologous pairing in species hybrids. Chromosoma 41, 63–73. 10.1007/BF00284074

[B44] Ezquerro-López D. Kopecký D. Aramendía L. (2017). Cytogenetic relationships within the Maghrebian clade of *Festuca* subgen. *Schedonorus* (*Poaceae*), using flow cytometry and FISH. Anales del Jardín Botánico Madrid 74, 1. 10.3989/ajbm.2455

[B45] Fan C. Luo J. Zhang S. Liu M. Li Q. Li Y. (2019). Genetic mapping of a major QTL promoting homoeologous chromosome pairing in a wheat landrace. Theor. Appl. Genet. 132, 2155–2166. 10.1007/s00122-019-03344-x 31016346

[B46] Feldman M. Levy A. (2005). Allopolyploidy – a shaping force in the evolution of wheat genomes. Cytogenet. Genome. Res. 109, 250–258. 10.1159/000082407 15753584

[B47] Feldman M. (1966a). The effect of chromosomes 5B, 5D and 5A on chromosomal pairing in *Triticum aestivum* . Proc. Natl. Acad. Sci. U.S.A. 55, 1447–1453. 10.1073/pnas.55.6.1447 5227663 PMC224341

[B48] Feldman M. (1966b). The mechanism regulating pairing in *Triticum timopheevii* . Wheat Inf. Serv. 21, 1–2.

[B49] Fussell C. P. (1987). “The Rabl orientation: a prelude to synapsis,” in Meiosis. Ed. Moens P. B. (Orlando: Academic Press), 275–299.

[B50] Gallo P. H. Micheletti P. L. Boldrini K. R. Risso-Pascotto C. Pagliarini M. S. do Valle C. B. (2007). 2n Gamete formation in the genus *Brachiaria* (*Poaceae: Paniceae*). Euphytica 154, 255–260. 10.1007/s10681-006-9294-1

[B51] Gauthier F. M. McGuinnis R. C. (1968). The meiotic behaviour of a nulli-haploid plant in *Avena* s*ativa* L. Can. J. Genet. Cytol. 10, 186–189. 10.1139/g68-025

[B52] Ghesquière M. Humphreys M. W. Zwierzykowski Z. (2010). ““Festulolium,” in Fodder Crops and Amenity Grasses,” in Handbook of Plant Breeding, vol. 5 . Eds. Boller B. Posselt U. Veronesi F. (New York, NY: Springer), 288–311. 10.1007/978-1-4419-0760-8_12

[B53] Gill K. S. Gill B. S. Endo T. R. Mukai Y. (1993). Fine physical mapping of *Ph1*, a chromosome pairing regulator gene in polyploid wheat. Genetics 134, 1231–1236. 8375657 10.1093/genetics/134.4.1231 PMC1205590

[B54] Gonzalo A. Lucas M. Charpentier C. Sandmann G. Lloyd A. Jenczewski E. (2019). Reducing *MSH4* copy number prevents meiotic crossovers between non-homologous chromosomes in *Brassica napus* . Nat. Commun. 10, 2354. 10.1038/s41467-019-10010-9 31142748 PMC6541637

[B55] Gray A. J. Benham P. E. M. Raybould A. F. (1990). ““Spartina anglica — the evolutionary and ecological background,”,” in Spartina anglica — A Research Review. Eds. Gray A. J. Benham P. E. M. (London, UK: Institute of Terrestrial Ecology, Natural Environment Research Council), 5–10.

[B56] Griffiths S. Sharp R. Foote T. N. Bertin I. Wanous M. Reader S. (2006). Molecular characterization of *Ph1* as a major chromosome pairing locus in polyploid wheat. Nature 439, 749–752. 10.1038/nature04434 16467840

[B57] Gupta P. K. Fedak G. (1985). Genetic control of meiotic chromosome pairing in polyploids in the genus *Hordeum* . Can. J. Genet. Cytol. 27, 515– 530. 10.1139/g85-077

[B58] Gyawali Y. Zhang W. Chao S. Xu S. Cai X. (2019). Delimitation of wheat *ph1b* deletion and development of *ph1b*-specific DNA markers. Theor. Appl. Genet. 132, 195–204. 10.1007/s00122-018-3207-2 30343385

[B59] Ha M. Lu J. Tian L. Ramachandran V. Kasschau K. D. Chapman E. J. (2009). Small RNAs serve as a genetic buffer against genomic shock in *Arabidopsis* interspecific hybrids and allopolyploids. Proc. Natl. Acad. Sci. U.S.A. 106, 17835–17840. 10.1073/pnas.0907003106 19805056 PMC2757398

[B60] Hajjar R. Hodgkin T. (2007). The use of wild relatives in crop improvement: a survey of developments over the last 20 years. Euphytica 156, 1–13. 10.1007/s10681-007-9363-0

[B61] Han C. Zhang P. Ryan P. R. Rathjen T. M. Yan Z. Delhaize E. (2016). Introgression of genes from bread wheat enhances the aluminium tolerance of durum wheat. Theor. Appl. Genet. 129, 729–739. 10.1007/s00122-015-2661-3 26747046

[B62] Hand M. L. Cogan N. O. Stewart A. V. Forster J. W. (2010). Evolutionary history of tall fescue morphotypes inferred from molecular phylogenetics of the *Lolium*-*Festuca* species complex. BMC Evol. Biol. 10, 303. 10.1186/1471-2148-10-303 20937141 PMC2958922

[B63] Hao M. Luo J. T. Yang M. Zhang L. Q. Yan Z. H. Yuan Z. W. (2011). Comparison of homoeologous chromosome pairing between hybrids of wheat genotypes Chinese Spring *ph1b* and Kaixian-luohanmai with rye. Genome 54, 959–964. 10.1139/g11-062 22070394

[B64] Harper L. Golubovskaya I. Cande W. Z. (2004). A bouquet of chromosomes. J. Cell Sci. 117, 4025–4032. 10.1242/jcs.01363 15316078

[B65] Holm P. B. Wang X. (1988). The effect of chromosome 5B on synapsis and chiasma formation in wheat, *Triticum aestivum* cv. Chin. spring. Carls. Res. Communs. 53, 191–208. 10.1007/BF02907179

[B66] Holm P. B. (1986). Chromosome pairing and chiasma formation in allohexaploid wheat, *Triticum aestivum* analyzed by spreading of meiotic nuclei. Carlsberg. Res. Commun. 51, 239. 10.1007/BF02906837

[B67] Huang S. Sirikhachornkit A. Su X. Faris J. Gill B. Haselkorn R. (2002). Genes encoding plastid acetyl-CoA carboxylase and 3-phosphoglycerate kinase of the *Triticum*/*Aegilops* complex and the evolutionary history of polyploid wheat. Proc. Natl. Acad. Sci. U.S.A. 99, 8133–8138. 10.1073/pnas.072223799 12060759 PMC123033

[B68] Hubbard J. C. E. (1968). Grasses. 2nd edn (London: Penguin Books).

[B69] Humphreys M. W. Thomas H. M. Morgan W. G. Meredith M. R. Harper J. A. Thomas A. (1995). Discriminating the ancestral progenitors of hexaploid *Festuca arundinacea* using genomic in situ hybridization. Heredity 75, 171–174. 10.1038/hdy.1995.120

[B70] Husband B. C. (2004). The role of triploid hybrids in the evolutionary dynamics of mixed-ploidy populations. Biol. J. Linn. Soc 82, 537–546. 10.1111/j.1095-8312.2004.00339.x

[B71] Jacobs B. F. Kingston J. D. Jacobs L. L. (1999). The origin of grass-dominated ecosystems. Ann. Mo. Bot. Gard. 86, 590–643. 10.2307/2666186

[B72] Jauhar P. P. Almouslem A. B. Peterson T. S. Joppa L. R. (1999). Inter- and intra-genomic chromosome pairing in haploids of durum wheat. J. Hered. 90, 437–445. 10.1093/jhered/90.4.437

[B73] Jauhar P. P. (1975). Genetic regulation of diploid-like chromosome pairing in the hexaploid species, *Festuca arundinacea* Schreb. and *F. rubra* L. (*Gramineae*). Chromosoma 52, 363–382. 10.1007/BF00364020

[B74] Jauhar P. P. (1977). Genetic regulation of diploid-like chromosome pairing in *Avena* . Theor. Appl. Genet. 49, 287–295. 10.1007/BF00275135 24407418

[B75] Jauhar P. P. (1993). Cytogenetics of the Festuca-Lolium complex: relevance to breeding (Berlin: Springer).

[B76] Jauhar P. P. (2003). Formation of 2n gametes in durum wheat haploids: sexual polyploidization. Euphytica 133, 81–94. 10.1023/A:1025692422665

[B77] Jenczewski E. Alix K. (2004). From diploids to allopolyploids: the emergence of efficient pairing control genes in plants. Crit. Rev. Plant Sci. 23, 21–45. 10.1080/07352680490273239

[B78] Jeon J. Chung Y. Lee S. Yi G. Oh B. An G. (1999). Isolation and characterization of an anther-specific gene, RA8, from rice (*Oryza sativa* L.). Plant Mol. Biol. 39, 35–44. 10.1023/A:1006157603096 10080707

[B79] Ji L. Langridge P. (1994). An early meiosis cDNA clone from wheat. Molec. Gen. Genet. 243, 17–23. 10.1007/BF00283871 8190067

[B80] Jiao Y. Wickett N. Ayyampalayam S. Chanderbali A. S. Landherr L. Ralph P. E. (2011). Ancestral polyploidy in seed plants and angiosperms. Nature 473, 97–100. 10.1038/nature09916 21478875

[B81] Kang H. Y. Zhang H. Q. Wang Y. Jiang Y. Yuan H. J. Zhou Y. H. (2008). Comparative analysis of the homoeologous pairing effects of *phKL* gene in common wheat × *Psathyrostachys huashanica* . Keng Cereal Res. Commun. 36, 429–440. 10.1556/CRC.36.2008.3.7

[B82] Keeler K. H. Davis G. A. (1999). Comparison of common cytotypes of *Andropogon gerardii* (*Andropogoneae*: Poaceae). Am. J. Bot. 86, 974–979. 10.2307/2656614 10406720

[B83] Keeler K. H. (1998). “Population biology of intraspecific polyploidy in grasses,” in Population Biology of Grasses. Ed. Cheplick G. P. (Cambridge, UK: Cambridge University Press), 183–206.

[B84] Kellogg E. A. (2001). Evolutionary history of the grasses. Plant Physiol. 125, 1198–1205. 10.1104/pp.125.3.1198 11244101 PMC1539375

[B85] King J. Grewal S. Yang C. Y. Hubbart S. Scholefield D. Ashling S. (2016). A step change in the transfer of interspecific variation into wheat from *Amblyopyrum muticum* . Plant Biotech. J. 15, 217–226. 10.1111/pbi.1206 PMC5258861 27459228

[B86] King J. Newell C. Grewal S. Hubbart-Edwards S. Yang C. Y. Scholefield D. (2019). Development of stable homozygous wheat/*Amblyopyrum muticum* (*Aegilops mutica*) introgression lines and their cytogenetic and molecular characterization. Front. Plant Sci. 10, 34. 10.3389/fpls.2019.00034 30792722 PMC6365885

[B87] Kleijer G. Morel P. (1984). Cytogenetic studies of crosses between *Lolium multiflorum* Lam. and *Festuca arundinacea* Schreb. II. The amphidiploids. Z. Pflanzenzücht 93, 23–42.

[B88] Koo D. Liu W. Friebe B. Gill B. S. (2016). Homoeologous recombination in the presence of *Ph1* gene in wheat. Chromosoma 126, 531–540. 10.1007/s00412-016-0622-5 27909815

[B89] Kopecký D. Loureiro J. Zwierzykowski Z. Ghesquière M. Doležel J. (2006). Genome constitution and evolution in *Lolium* × *Festuca* hybrid cultivars (Festulolium). Theor. Appl. Genet. 113, 731–742. 10.1007/s00122-006-0341-z 16832647

[B90] Kopecký D. Lukaszewski A. J. Doležel J. (2008a). Meiotic behaviour of individual chromosomes of *Festuca pratensis* in tetraploid *Lolium multiflorum* . Chromosome Res. 16, 987. 10.1007/s10577-008-1256-0 18830677

[B91] Kopecký D. Lukaszewski A. J. Doležel J. (2008b). Cytogenetics of Festulolium (*Festuca* x *Lolium* hybrids). Cytogenet. Genome Res. 120, 370–383. 10.1159/000121086 18504366

[B92] Kopecký D. Bartoš J. Zwierzykowski Z. Doležel J. (2009). Chromosome pairing of individual genomes in tall fescue (*Festuca arundinacea* Schreb.), its progenitors, and hybrids with Italian ryegrass (*Lolium multiflorum* Lam.). Cytogenet. Genome Res. 124, 170–178. 10.1159/000207525 19420930

[B93] Kopecký D. Talukder S. K. Zwyrtková J. Trammell M. Doležel J. Saha M. C. (2019). Inter-morphotype hybridization in tall fescue (*Festuca arundinacea* Schreb.): exploration of meiotic irregularities and potential for breeding. Euphytica 215, 97. 10.1007/s10681-019-2419-0

[B94] Kreiner J. M. Kron P. Husband B. C. (2017). Evolutionary dynamics of unreduced gametes. Trends Genet. 33, 583–593. 10.1016/j.tig.2017.06.009 28732599

[B95] Ladizinsky G. (1973). Genetic control of bivalent pairing in the *Avena strigosa* polyploid complex. Chromosoma 42, 105–110. 10.1007/BF00326334 4712856

[B96] Leggett J. M. Thomas H. (1995). “Oat evolution and cytogenetics,” in The Oat Crop. World Crop Series. Ed. Welch R. W. (Dordrecht, DE: Springer), 120–149. 10.1007/978-94-011-0015-1_5

[B97] Li H. Deal K. R. Luo M. C. Ji W. Distelfeld A. Dvorak J. (2017). Introgression of the *Aegilops speltoides Su1-Ph1* Suppressor into Wheat. Front. Plant Sci. 8, 2163. 10.3389/fpls.2017.02163 29326749 PMC5742420

[B98] Liu D. C. Luo M. C. Yang J. L. Yen C. Lan X. J. Yang W. Y. (1997). Chromosome location of a new paring promoter in natural populations of common wheat. Xi Nan Nong Ye Xue Bao 10, 10–15. (in Chinese).

[B99] Liu D. C. Luo M. C. Yen C. Yang J. L. Yang W. Y. (1998). “The promotion of homoeologous pairing in hybrids of common wheat cv. Kaixianluohanmai with alien species,” in Proceedings of the 9th International Wheat Genetics Symposium, vol. 4 . Ed. Slinkard A. E. (Saskatoon, CA: University Extension Press, University of Saskatchewan), 377–378.

[B100] Liu D. C. Zheng Y. L. Yan Z. H. Zhou Y. H. Wei Y. M. Lan X. J. (2003). Combination of homoeologous pairing gene *phKL* and *Ph2*-deficiency in common wheat and its meiotic behaviors in hybrids with alien species. Acta Bot. Sin. 45, 1121–1128.

[B101] Liu W. Rouse M. Friebe B. Jin Y. Gill B. S. Pumphrey M. O. (2011). Discovery and molecular mapping of a new gene conferring resistance to stem rust, *Sr53*, derived from *Aegilops geniculata* and characterization of spontaneous translocation stocks with reduced alien chromatin. Chromosom Res. 19, 669–682. 10.1007/s10577-011-9226-3 21728140

[B102] Lloyd A. Bomblies K. (2016). Meiosis in autopolyploid and allopolyploid *Arabidopsis* . Curr. Opin. Plant Biol. 30, 116–122. 10.1016/j.pbi.2016.02.004 26950252

[B103] Lloyd A. Ranoux M. Vautrin S. Glover N. M. Fourment J. Charif D. (2014). Meiotic gene evolution: can you teach a new dog new tricks? Mol. Biol. Evol. 31, 1724–1727. 10.1093/molbev/msu119 24694832

[B104] Loidl J. (1990). The initiation of meiotic chromosome pairing: the cytological view. Genome 33, 759–778. 10.1139/g90-115 2086352

[B105] Loureiro J. Kopecký D. Castro S. Santos C. Silveira P. (2007). Flow cytometric and cytogenetic analyses of Iberian Peninsula *Festuca* spp. Plant Syst. Evol. 269, 89–105. 10.1007/s00606-007-0564-8

[B106] Lukaszewski A. J. Kopecký D. (2010). The *ph1* locus from wheat controls meiotic chromosome pairing in autotetraploid rye (*Secale cereale* L.). Cytogenet. Genome Res. 129, 117–123. 10.1159/000314279 20551609

[B107] Luo M. C. Yang Z. L. Yen C. Yang J. L. (1992). ““The cytogenetic investigation on F1 hybrid of Chinese wheat landrace,”,” in Exploration of Crop Breeding. Eds. Ren Z. L. Peng J. H. (Sichuan: Science and Technology Press), 169–176.

[B108] Maestra B. Naranjo T. (1997). Homoeologous relationships of *Triticum sharonense* chromosomes to *T.* *aestivum* . Theor. Appl. Genet. 94, 657–663. 10.1007/s001220050463

[B109] Maestra B. Naranjo T. (1998). Homoeologous relationships of *Aegilops speltoides* chromosomes to bread wheat. Theor. Appl. Genet. 97, 181–186. 10.1007/s001220050883

[B110] Malik C. P. Thomas P. T. (1967). Cytological relationships and genome structure of some *Festuca* species. Caryologia 20, 1–39. 10.1080/00087114.1967.10796244

[B111] Marais G. F. Marais A. S. Eksteen A. Pretorius Z. A. (2010). Modification of the *Aegilops neglecta*-common wheat *Lr62*/*Yr42* translocation through allosyndetic pairing induction. Crop Sci. 49, 871–879. 10.2135/cropsci2008.06.0317

[B112] Marcussen T. Sandve S. R. Heier L. Spannagl M. Pfeifer M. IWGSC (2014). Ancient hybridizations among the ancestral genomes of bread wheat. Science 345, 6194. 10.1126/science.1250092 25035499

[B113] Marques A. M. Tuler A. C. Carvalho C. R. Carrijo T. T. Ferreira M. F. Clarindo W. R. (2016). Refinement of the karyological aspects of *Psidium guineense* (Swartz 1788): a comparison with *Psidium guajava* (Linnaeus 1753). Comp. Cytogenet. 10, 117–128. 10.3897/CompCytogen.v10i1.6462 27186342 PMC4856930

[B114] Martín A. C. Shaw P. Phillips D. Reader S. Moore G. (2014). Licensing *MLH1* sites for crossover during meiosis. Nat. Commun. 5, 1–5. 10.1038/ncomms5580 PMC4143925 25098240

[B115] Martín A. C. Rey M. D. Shaw P. Moore G. (2017). Dual effect of the wheat *Ph1* locus on chromosome synapsis and crossover. Chromosoma 126, 669–680. 10.1007/s00412-017-0630-0 28365783 PMC5688220

[B116] Martínez M. Cuñado N. Carcelén N. Romero C. (2001a). The *Ph1* and *Ph2* loci play different roles in the synaptic behaviour of hexaploid wheat *Triticum aestivum* . Theor. Appl. Genet. 103, 398–405. 10.1007/s00122-001-0543-3

[B117] Martínez M. Naranjo T. Cuadrado C. Romero C. (2001b). The synaptic behaviour of *Triticum turgidum* with variable doses of the *Ph1* locus. Theor. Appl. Genet. 102, 751–758. 10.1007/s001220051706

[B118] Martínez-Pérez E. Shaw P. Reader S. Aragón-Alcaide L. Miller T. Moore G. (1999). Homologous chromosome pairing in wheat. J. Cell Sci. 112, 1761–1769. 10318768 10.1242/jcs.112.11.1761

[B119] Mason A. S. Pires J. C. (2015). Unreduced gametes: meiotic mishap or evolutionary mechanism? Trends Genet. 31, 5–10. 10.1016/j.tig.2014.09.011 25445549

[B120] Mason A. S. Nelson M. N. Yan G. Cowling W. A. (2011). Production of viable male unreduced gametes in *Brassica* interspecific hybrids is genotype specific and stimulated by cold temperatures. BMC Plant Biol. 11:, 103. 10.1186/1471-2229-11-103 21663695 PMC3141635

[B121] McGuire P. E. Dvořák J. (1982). Genetic regulation of heterogenetic chromosome pairing in polyploid species of the genus Triticum sensu lato. Can. J. Genet. Cytol. 24, 57–82. 10.1139/g82-007

[B122] Meister N. Tjumjakoff N. A. (1928). Rye–wheat hybrids from reciprocal crosses. J. Genet. 20, 233–245. 10.1007/BF02983142

[B123] Mello-Sampayo T. Canas A. P. (1973). “Suppression of meiotic chromosome pairing in common wheat,” in Proceedings of the 4th International Wheat Genetics Symposium. Eds. Sears E. R. ER L. M. S. (Columbia, MI: Agricultural Experiment Station, College of Agriculture, University of Missouri), 703–713.

[B124] Mello-Sampayo T. (1968). ““Homoeologous chromosome pairing in pentaploid hybrids of wheat,”,” in Third International Wheat Genetics Symposium. Eds. Finlay K. W. Shepherd K. W. (Canberra: Butterworth & Company), 179–184.

[B125] Mello-Sampayo T. (1971). Genetic regulation of meiotic chromosome pairing by chromosome-3D of *Triticum aestivum* . Nat. New Biol. 230, 22. 10.1038/newbio230022a0 5283628

[B126] Meyers L. A. Levin D. A. (2006). On the abundance of polyploids in flowering plants. Evolution 60, 1198–1206. 10.1111/j.0014-3820.2006.tb01198.x 16892970

[B127] Mikhailova E. I. Naranjo T. Shepherd K. Wennekes-van E. J. Heyting C. de Jong H. (1998). The effect of the wheat *Ph1* locus on chromatin organisation and meiotic pairing analysed by genome painting. Chromosoma 107, 339–350. 10.1007/s004120050316 9880767

[B128] Murat F. Zhang R. Guizard S. Flores R. Armero A. Pont C. (2014). Shared subgenome dominance following polyploidization explains grass genome evolutionary plasticity from a seven protochromosome ancestor with 16K protogenes. Genome Biol. Evol. 6, 12–33. 10.1093/gbe/evt200 24317974 PMC3914691

[B129] Murray B. G. Sieber V. K. Jackson R. C. (1984). Further evidence for the presence of meiotic pairing control genes in *Alopecurus* L. (Gramineae). Genet. 63, 13–20. 10.1007/BF00137460

[B130] Naranjo T. Maestra B. (1995). The effect of ph mutations on homoeologous pairing in hybrids of wheat with *Triticum longissimum* . Theor. Appl. Genet. 91, 1265–1270. 10.1007/BF00220939 24170056

[B131] Naranjo T. Roca A. Goicoechea P. G. Giráldez R. (1987). Arm homoeology of wheat and rye chromosomes. Genome 29, 873–882. 10.1139/g87-149

[B132] Naranjo T. Roca A. Goicoechea P. G. Giráldez R. (1988). “Chromosome structure of common wheat: genome reassignment of chromosomes 4A and 4B,” in Proceedings of the 7th International Wheat Genetics Symposium, eds. Eds. Miller T. E. Koebner R. M. D. (Cambridge, UK: Cambridge University), 115–120.

[B133] Naranjo T. (2015). Contribution of Structural Chromosome Mutants to the Study of Meiosis in Plants. Cytogenet. Genome Res. 147, 55–69. 10.1159/000442219 26658116

[B134] Naranjo T. (2019). The effect of chromosome structure upon meiotic homologous and homoeologous recombinations in *Triticeae* . Agronomy 9, 552. 10.3390/agronomy9090552

[B135] Niu Z. Klindworth D. L. Friesen T. L. Chao S. Jin Y. Cai X. (2011). Targeted introgression of a wheat stem rust resistance gene by DNA marker-assisted chromosome engineering. Genetics 187, 1011–1021. 10.1534/genetics.110.123588 21242535 PMC3070511

[B136] Ortega S. Prieto I. Odajima J. Martín A. Dubus P. Sotillo R. (2003). Cyclin-dependent kinase 2 is essential for meiosis but not for mitotic cell division in mice. Nat. Genet. 35, 25–31. 10.1038/ng1232 12923533

[B137] Osborn T. C. Pires J. C. Birchler J. A. Auger D. L. Chen Z. J. Lee H. S. (2003). Understanding mechanisms of novel gene expression in polyploids. Trends Genet. 19, 141–147. 10.1016/S0168-9525(03)00015-5 12615008

[B138] Otto S. P. Whitton J. (2000). Polyploid incidence and evolution. Annu. Rev. Genet. 34, 401–437. 10.1146/annurev.genet.34.1.401 11092833

[B139] Ozkan H. Feldman M. (2001). Genotypic variation in tetraploid wheat affecting homoeologous pairing in hybrids with *Aegilops peregrina* . Genome 44, 1000–1006. 10.1139/g01-100 11768203

[B140] Pécrix Y. Rallo G. Folzer H. Cigna M. Gudin S. Le Bris M. (2011). Polyploidization mechanisms: temperature environment can induce diploid gamete formation in *Rosa* sp. J. Exp. Bot. 62, 3587–3597. 10.1093/jxb/err052 21398431

[B141] Pecinka A. Fang W. Rehmsmeier M. Levy A. A. Scheid O. M. (2011). Polyploidization increases meiotic recombination frequency in *Arabidopsis* . BMC Biol. 9, 24. 10.1186/1741-7007-9-24 21510849 PMC3110136

[B142] Pelé A. Rousseau-Gueutin M. Chèvre A. M. (2018). Speciation success of polyploid plants closely relates to the regulation of meiotic recombination. Front. Plant Sci. 9, 907. 10.3389/fpls.2018.00907 30002669 PMC6031745

[B143] Pernickova K. Linc G. Gaal E. Kopecký D. Šamajová O. Lukaszewski A. (2019). Out-of-position telomeres in meiotic leptotene appear responsible for chiasmate pairing in an inversion heterozygote in wheat (*Triticum aestivum* L.). Chromosoma 128, 31–39. 10.1007/s00412-018-0686-5 30483879

[B144] Petersen G. Seberg O. Yde M. Berthelsen K. (2006). Phylogenetic relationships of *Triticum* and *Aegilops* and evidence for the origin of the A, B, and D genomes of common wheat (*Triticum aestivum*). Mol. Phylogenet. Evol. 39, 70–82. 10.1016/j.ympev.2006.01.023 16504543

[B145] Prieto P. Moore G. Reader S. (2005). Control of conformation changes associated with homologue recognition during meiosis. Theor. Appl. Genet. 111, 505–510. 10.1007/s00122-005-2040-6 15895201

[B146] Ramsey J. Schemske D. W. (1998). Pathways, mechanisms, and rates of polyploid formation in flowering plants. Annu. Rev. Ecol. Syst. 29, 467–501. 10.1146/annurev.ecolsys.29.1.467

[B147] Ramsey J. Schemske D. W. (2002). Neopolyploidy in flowering plants. Annu. Rev. Ecol. Syst. 33, 589–639. 10.1146/annurev.ecolsys.33.010802.150437

[B148] Renny-Byfield S. Rodgers-Melnick E. Ross-Ibara J. (2017). Gene fractionation and function in the ancient subgenomes of maize. Mol. Biol. Evol. 34, 1825–1832. 10.1093/molbev/msx121 28430989

[B149] Rey M. D. Calderón M. C. Prieto P. (2015a). The use of the *ph1b* mutant to induce recombination between the chromosomes of wheat and barley. Front. Plant Sci. 6, 160. 10.3389/fpls.2015.00160 25852713 PMC4365720

[B150] Rey M. D. Calderón M. C. Rodrigo M. J. Zacarías L. Alós E. Prieto P. (2015b). Novel Bread Wheat Lines Enriched in Carotenoids Carrying Hordeum chilense Chromosome Arms in the *ph1b* Background. PloS One 10 (8), e0134598. 10.1371/journal.pone.0134598 26241856 PMC4524710

[B151] Rey M. Martín A. C. Higgins J. Swarbreck D. Uauy C. Shaw P. (2017). Exploiting the *ZIP4* homologue within the wheat *Ph1* locus has identified two lines exhibiting homoeologous crossover in wheat-wild relative hybrids. Mol. Breed. 37, 95. 10.1007/s11032-017-0700-2 28781573 PMC5515957

[B152] Rey M. D. Martin A. C. Smedley M. Hayta S. Harwood W. Shaw P. (2018). Magnesium increases homoeologous crossover frequency during meiosis in *ZIP4* (*Ph1* gene) mutant wheat-wild relative hybrids. Front. Plant Sci. 9, 509. 10.3389/fpls.2018.00509 29731763 PMC5920029

[B153] Riley R. Chapman V. (1958). Genetic control of the cytologically diploid behavior of hexaploid wheat. Nature 182, 713–715. . 10.1038/182713a0

[B154] Riley R. Kempanna C. (1963). The homoeologous nature of the non-homologous meiotic pairing in *Triticum aestivum* deficient for chromosome V. Heredity 18, 287–306. 10.1038/hdy.1963.31

[B155] Riley R. Law C. N. (1965). Genetic variation in chromosome pairing. Adv. Genet. 13, 57–114. 10.1016/S0065-2660(08)60047-4

[B156] Riley R. Chapman V. Miller T. E. (1973). “The determination of meiotic chromosome pairing,” in Proceedings of the 4th International Wheat Genetics Symposium. Eds. Sears E. R. ER L. M. S. (Columbia, MI: Agricultural Experiment Station, College of Agriculture, University of Missouri), 731–738.

[B157] Riley R. (1960). The diploidization of polyploid wheat. Heredity 15, 407–429. 10.1038/hdy.1960.106

[B158] Roberts M. A. Reader S. M. Dalgliesh C. Miller T. E. Foote T. N. Fish L. J. (1999). Induction and characterisation of the *Ph1* wheat mutants. Genetics 153, 1909–1918. 10581295 10.1093/genetics/153.4.1909 PMC1460846

[B159] Salmon A. Ainouche M. L. Wendel J. F. (2005). Genetic and epigenetic consequences of recent hybridization and polyploidy in *Spartina* (Poaceae). Mol. Ecol. 14, 1163–1175. 10.1111/j.1365-294X.2005.02488.x 15773943

[B160] Sánchez-Morán E. Benavente E. Orellana J. (2001). Analysis of karyotypic stability of homoeologous-pairing (*ph*) mutants in allopolyploid wheats. Chromosoma 110, 371–377 (2001). 10.1007/s004120100156 11685537

[B161] Sattler M. C. Carvalho C. R. Clarindo W. R. (2016). The polyploidy and its key role in plant breeding. Planta 243, 281–296. 10.1007/s00425-015-2450-x 26715561

[B162] Scherthan H. (2001). A bouquet makes ends meet. Nat. Rev. Mol. Cell Biol. 2, 621–627. 10.1038/35085086 11483995

[B163] Scherthan H. (2007). Telomere attachment and clustering during meiosis. Cell Mol. Life Sci. 64, 117–124. 10.1007/s00018-006-6463-2 17219025 PMC11136177

[B164] Schwarzacher T. (1997). Three stages of meiotic homologous chromosome pairing in wheat: cognition, alignment and synapsis. Sex- Plant Reprod. 10, 324–331. 10.1007/s004970050106

[B165] Sears E. R. Okamoto M. (1958). “Intergenomic chromosome relationship in hexaploid wheat,” in Proceedings of 10th International Congress of Genetics (Toronto, CA: University of Toronto Press), 258–259.

[B166] Sears E. R. (1976). Genetic control of chromosome pairing in wheat. Annu. Rev. Genet. 10, 31–51. 10.1146/annurev.ge.10.120176.000335 797311

[B167] Sears E. R. (1977). An induced mutant with homoeologous pairing in common wheat. Can. J. Genet. Cytol. 19, 585–593. 10.1139/g77-063

[B168] Sears E. R. (1982). A wheat mutation conditioning an intermediate level of homoeologous chromosome pairing. Can. J. Genet. Cytol. 24, 715–719. 10.1139/g82-076

[B169] Sears E. R. (1984). “Mutations in wheat that raise the level of meiotic chromosome pairing,” in Gene Manipulation in Plant Improvement. Proc. 16th Stadler Genet. Symp. Ed. Gustafson J. P. (New York, NY: Plenum Press), 295–300.

[B170] Shang X. M. Jackson R. C. NGuyen H. T. Huang H. T. (1989). Chromosome pairing in the *Triticum monococcum* complex: evidence for pairing control genes. Genome 32, 213–226. 10.1139/g89-432

[B171] Shen Y. Tang D. Wang K. Wang M. Huang J. Luo W. (2012). *ZIP4* in homologous chromosome synapsis and crossover formation in rice meiosis. J. Cell Sci. 125, 2581–2591. 10.1242/jcs.090993 22393242

[B172] Soltis D. E. Soltis P. S. Schemske D. W. Hancock J. F. Thompson J. N. Husband B. C. (2007). Autopolyploidy in angiosperms: have we grossly underestimated the number of species? Taxon 56, 13–30. 10.2307/25065732

[B173] Soreng R. J. Peterson P. M. Romaschenko K. Davidse G. Zuloaga F. O. Judziewicz E. J. (2015). A worldwide phylogenetic classification of the Poaceae (Gramineae). J. Syst. Evol. 53, 117–137. 10.1111/jse.12150

[B174] Stebbins G. L. (1950). Variation and Evolution in Plants (New York: Columbia University Press).

[B175] Stebbins G. L. (1971). Chromosomal Evolution in Higher Plants (London: Addison-Wesley).

[B176] Sun Y. Wu Y. Yang C. Sun S. Lin X. Liu L. (2017). Segmental allotetraploidy generates extensive homoeologous expression rewiring and phenotypic diversity at the population level in rice. Mol. Ecol. 26, 5451–5466. 10.1111/mec.14297 28802080

[B177] Sutton T. Whitford R. Baumann U. Dong C. M. Able J. A. Langridge P. (2003). The *Ph2* pairing homoeologous locus of wheat (*Triticum aestivum*): identification of candidate meiotic genes using a comparative genetics approach. Plant J. 36, 443–456. 10.1046/j.1365-313X.2003.01891.x 14617076

[B178] Svačina R. Karafiátová M. Malurová M. Serra H. Vítek D. Endo T. R. (2020). Development of deletion lines for chromosome 3D of bread wheat. Front. Plant Sci. 10, 1756. 10.3389/fpls.2019.01756 32047508 PMC6997527

[B179] Tamayo-Ordóñez M. C. Espinosa-Barrera L. A. Tamayo-Ordóñez Y. J. Ayil-Gutiérrez B. Sánchez-Teyer L. F. (2016). Advances and perspectives in the generation of polyploid plant species. Euphytica 209, 1–22. 10.1007/s10681-016-1646-x

[B180] Thomas H. M. Morgan W. G. Meredith M. R. Humphreys M. W. Leggett J. M. (1994). Identification of parental and recombined chromosomes in hybrid derivatives of *Lolium multiflorum* × *Festuca pratensis* by genomic in situ hybridization. Theor. Appl. Genet. 88, 909–913. 10.1007/BF00220795 24186241

[B181] Thomas H. (1992). “Cytogenetics of Avena,” in Oat Science and Technology. Monograph 33, Agronomy Series. Eds. Marshall H. G. Sorrells M. E. (Madison, WI: ASA and CSSA), 473–507.

[B182] Thomas S. W. (1997). Molecular studies of homologous chromosome pairing in *Triticum aestivum* . [dissertation]. [Adelaide]: University of Adelaide.

[B183] Thompson J. D. McNeilly T. Gray A. J. (1991). Population variation in *Spartina anglica* C.E. Hubbard. I. Evidence from a common garden experiment. New Phytol. 117, 115–128. 10.1111/j.1469-8137.1991.tb00951.x

[B184] Van de Peer Y. Maere S. Meyer A. (2009). The evolutionary significance of ancient genome duplications. Nat. Rev. Genet. 10, 725–732. 10.1038/nrg2600 19652647

[B185] Van de Peer Y. Mizrachi E. Marchal K. (2017). The evolutionary significance of polyploidy. Nat. Rev. Genet. 18, 411–424. 10.1038/nrg.2017.26 28502977

[B186] Vanneste K. Baele G. Maere S. Van de Peer Y. (2014). Analysis of 41 plant genomes supports a wave of successful genome duplications in association with the Cretaceous–Paleogene boundary. Genome Res. 24, 1334–1347. 10.1101/gr.168997.113 24835588 PMC4120086

[B187] Viegas W. S. Mello-Sampayo T. Feldman M. Avivi L. (1980). Reduction of chromosome pairing by a spontaneous mutation on chromosomal arm 5DL of *Triticum aestivum* . Can. J. Genet. Cytol. 22, 569–575. 10.1139/g80-062

[B188] Viera A. Rufas J. S. Martinez I. Barbero J. L. Ortega S. Suja J. (2009). CDK2 is required for proper homologous pairing, recombination and sex-body formation during male meiosis. J. Cell Sci. 122, 2149–2159. 10.1242/jcs.046706 19494131

[B189] Villar R. Veneklaas E. J. Jordano P. Lambers H. (1998). Relative growth rate and biomass allocation in 20 *Aegilops* (Poaceae) species. N. Phytol. 140, 425–437. 10.1046/j.1469-8137.1998.00286.x 33862869

[B190] von Well E. Fossey A. (1998). A comparative investigation of seed germination, metabolism and seedling growth between two polyploid *Triticum* species. Euphytica 101, 83–89. 10.1023/A:1018320230154

[B191] Waines J. G. (1976). A model for the origin of diploidizing mechanisms in poly-ploid species. Am. Nat. 110, 415– 430. 10.1086/283077

[B192] Wall A. M. Riley R. Chapman V. (1971). Wheat mutants permitting homoeologous meiotic chromosomes pairing. Genet. Res. 18, 311–328. 10.1017/S0016672300012714

[B193] Wang A. Xia Q. Xie W. Datla R. Selvaraj G. (2003). The classical Ubisch bodies carry a sporophytically produced structural protein (RAFTIN) that is essential for pollen development. Proc. Natl. Acad. Sci. U.S.A. 100, 14487–14492. 10.1073/pnas.2231254100 14612572 PMC283618

[B194] Wang J. Roe B. Macmil S. Yu Q. Murray J. E. Tang H. (2010). Microcollinearity between autopolyploid sugarcane and diploid sorghum genomes. BMC Genomics 11, 261. 10.1186/1471-2164-11-261 20416060 PMC2882929

[B195] Whitford R. (2002). From intimate chromosome associations to wild sex in wheat (*Triticum aestivum*). [dissertation]. [Adelaide]: University of Adelaide.

[B196] Winterfeld G. Schneider J. Perner K. Röser M. (2012). Origin of highly polyploids: different pathways of auto- and allopolyploidy in 12–18*x* species of *Avenula* (Poaceae). Int. J. Pl. Sci. 173, 1–14. 10.1086/664710

[B197] Wulff B. B. H. Moscou M. J. (2014). Strategies for transferring resistance into wheat: from wide crosses to GM cassettes. Front. Plant Sci. 5, 692. 10.3389/fpls.2014.00692 25538723 PMC4255625

[B198] Xiang Z. G. Liu D. C. Zheng Y. L. Zhang L. Q. Yan Z. H. (2005). The effect of *phKL* gene on homoeologous pairing of wheat-alien hybrids is situated between gene mutants of *Ph1* and *Ph2* . Hereditas 27, 935–940. 16378942

[B199] Xiong Y. G. Gan L. Hu Y. P. Sun W. C. Zhou X. Song Z. J. (2019). *OsMND1* regulates early meiosis and improves the seed set rate in polyploid rice. Plant Growth Regul. 87, 341–356. 10.1007/s10725-019-00476-4

[B200] Yant L. Hollister J. D. Wright K. M. Arnold B. J. Higgins J. D. Franklin F. C. H. (2013). Meiotic adaptation to genome duplication in *Arabidopsis arenosa* . Curr. Biol. 23, 2151–2156. 10.1016/j.cub.2013.08.059 24139735 PMC3859316

